# A case of behavioural diversification in male floral function – the evolution of thigmonastic pollen presentation

**DOI:** 10.1038/s41598-018-32384-4

**Published:** 2018-09-19

**Authors:** Tilo Henning, Moritz Mittelbach, Sascha A. Ismail, Rafael H. Acuña-Castillo, Maximilian Weigend

**Affiliations:** 1 Botanic Garden and Botanical Museum Berlin-Dahlem, Freie Universität Berlin, Königin-Luise-Str. 6-8, 14195 Berlin, Germany; 2 Institute of Biologie, Freie Universität Berlin, Altensteinstr. 6, 14195 Berlin, Germany; 3 School of Biological Sciences, University of Aberdeen, 23 St. Machar Drive, Aberdeen, AB24 3UU Scotland; 4 Nees Institut für Biodiversität der Pflanzen, Rheinische Friedrich-Wilhelms-Universität Bonn, Meckenheimer Allee 170, 53115 Bonn, Germany; 5 Universidad de Costa Rica, Escuela de Biología, Apdo, Postal: 11501-2060, San Pedro de Montes de Oca, Costa Rica

## Abstract

Obvious movements of plant organs have fascinated scientists for a long time. They have been studied extensively, but few behavioural studies to date have dealt with them, and hardly anything is known about their evolution. Here, we present a large experimental dataset on the stamen movement patterns found in the Loasaceae subfam. Loasoideae (Cornales). An evolutionary transition from autonomous-only to a combination of autonomous and thigmonastic stamen movement with increased complexity was experimentally demonstrated. We compare the stamen movement patterns with extensive pollinator observations and discuss it in the context of male mating behavior. Thigmonastic pollen presentation via stamen movements appears to be a crucial component of floral adaptation to pollinator behaviour, evolving in concert with complex adjustments of flower signal, reward and morphology. We hypothesize that rapid adjustments of pollen presentation timing may play a significant role in the diversification of this plant group, representing a striking example for the evolutionary significance of plant behaviour.

## Introduction

### Plant behaviour

Plant behavioural studies are gradually being accepted as a branch of plant science^1–4^. Numerous aspects of plant intelligence, including neurobiology and behavioural responses dealing with biotic and abiotic stimuli, have been invoked to describe and explain complex reactions of plants to stimuli. Only recently have studies documented plant learning^5^ and even discussed visual cognition^6,7^. Without trying to summarize the numerous aspects of plant behaviour that have been described in recent years^1,4,8^, it is clear that plants have long been perceived as passive organisms.

Most scientific evidence on plant behaviour circumscribes individual phenomena or compares distantly related taxa, missing a possible linkage between behaviour and evolutionary processes^9^. Behaviour is fundamental for understanding the fitness of an individual organism, as has been amply documented in the animal kingdom, but it also conveys competitive advantages at the population and meta-population level and is thus instrumental in driving natural selection. Behavioural diversification has long been known to be a driver of diversification in the animal kingdom (e.g. birds^10^; poison frogs – *Oophaga granulifera*^11^). Behavioural isolation, often concerning mating behaviour, has been instrumental in circumscribing animal species, (crabs – *Uca* sp^12–16^). The potential evolutionary implications of plant behaviour, however, have not yet been studied – since there are few known examples and previous investigations have focused on individual species, and such investigations precluded any comparative or phylogenetic analyses.

### Stamen Movement

The active movement of plant organs, in particular those that are fast and therefore obvious, have fascinated scientists ever since their first discovery^17,18^. Rapid movements of specialized organs have been studied quite extensively, e.g., the trap mechanisms of *Dionaea muscipula* or *Aldrovanda vesiculosa*^19^ or the leaf movements of *Mimosa pudica*^20^ or *Albizzia julibrissin*^21^. These movements serve to protect the plant body from physical damage or to catch animal prey for plant nutrition. Conversely, a wide spectrum of less obvious movements of floral organs can be observed in the context of pollination ecology. Among these, stamen movements are the most common type and have been reported from a range of plant families (see^19^). Stamen movements have been known for a long time (*Berberis* – Berberidaceae^22^; *Parietaria* – Urticaceae^23^). The functional interrelation between these movements and flower visitors^23^ and the process of pollination (*Nigella* – Ranunculaceae^24^) was reported as early as the 19^th^ century. Several more or less spectacular cases of stamen movements have been reported from a variety of plant families. These movements are either singular movements driven by unrepeatable releases of stored energy (e.g. *Ricinus* – Euphorbiaceae^25^; *Trophis* – Moraceae^26,27^; *Catasetum* – Orchidaceae^28^; *Cornus canadensis* – Cornaceae^29^), or are slower, cascade-like movements that lead to the consecutive movement of stamens within the flower (*Tropaeolum* – Tropaeolaceae and *Parnassia* – Celastraceae^30^) or the movement can be repeatedly triggered by flower visitors (e.g. *Berberis*^31^). For *Ruta graveolens* (Rutaceae), Ren and Tang^32^ revealed a combination of an autonomous, successive movement complemented by an accelerated stamen uplift triggered by an increased number of pollinator visits on the flower. Such thigmonastic stamen movements (thigmonasty = nastic response to touch or vibration – in stamens = triggered by the contact with flower visitors) have been reported for several plant families: Aizoaceae, Berberidaceae, Cactaceae, Cistaceae, Malvaceae, Portulacaceae, and Tiliaceae^33–38^ but are often restricted to a single taxon. The majority of these movements follow uniform patterns: a single stimulus leads to the simultaneous, unrepeatable movement of all stamens in a fixed direction in order to achieve maximum pollen deposition on a pollinator. In almost all cases, this movement is triggered by stimulating the stamen (usually at the filament). Only few examples of more complex responses have been reported. In *Stylidium* (Stylidiaceae) the stamens and style form a columnar complex that can perform repeated rapid movements from one side of the flower to the other^39^. In *Berberis*, the intensity of the stimulus determines the number of stamens that move in response^22^ and in *Opuntia lindheimeri*, the direction of the movement is determined by the exact location of the stimulus^40^. In many other species of *Opuntia* the stamens, upon stimulation, repeatably perform a bidirectional movement from the petals towards the style and back, regardless of the specific site of contact^41,42^. Finally, cascade movement mechanisms (whether thigmonastic or not) often occur in combination with subsequent autonomous movements to avoid anther-anther interference during pollination (e.g. *Parnassia*^43^; *Ruta graveolens*^32^). Moreover, in the latter case all stamens repeat their movement towards the style simultaneously at the end of anthesis to ensure pollination through selfing as a backup mechanism^32^.

Members of the Loasaceae subfam. Loasoideae have an even more complex stamen presentation. Sequentially maturing stamens individually move into the centre of the flower, ancestrally this movement appears to be exclusively autonomous, but in the derived condition appears to be triggered and thigmonastic^44^. Unlike in most other plants with thigmonastic stamens, the stimulation does not lead to the indiscriminate movement of all, or multiple stamens, but only a small and relatively fixed number of stamens reacts to each stimulus^44–47^. Individual stamens can be triggered throughout the staminate phase for as long as fresh stamens are available. Finally, the mechanical stimulus is not received by the stamen itself, but by the so-called nectar scales (see below). Flower visitors manipulate these scales in order to access the nectar and this stimulus is transmitted to the stamen fascicles, linking actual nectar harvest to pollen dispensation^47^. The stimulus thus has to be transmitted through the receptacle from the nectar scale to the stamen. This remarkably complex mechanism has been widely documented for representatives of subfam. Loasoideae, but nowhere else in the plant kingdom^46^. In Loasaceae, this reaction is one aspect of the considerable diversification of floral morphology and function. It has been argued that thigmonastic stamen presentation is a highly specialized case of pollen partitioning and a mechanism to increase male fitness, and data have been presented indicating that the specific timing of pollen presentation is likely to increase pollen export^45^.

The stamen movement observed in Loasaceae subfam. Loasoideae is in line with the predictions made in the context of the pollen-presentation theory^48,49^: Plants can increase male fitness by adjusting pollen presentation to pollinator quality and quantity. If the mechanism of pollen presentation adjusts to a certain pollinator’s traplining behaviour and makes use of pollinator revisits, then outbreeding success would likely increase. LeBuhn and Holsinger (p. 119^50^) concluded that: “A plant should allocate pollen such that all pollinators that visit remove pollen”. Such a system of pollen packaging and dispensing would require either a very constant frequency of revisits or a mode of pollen presentation that can adjust to the pollinator activity. LeBuhn and Holsinger (p. 119–120^50^) called this the “unlikely case in which the number of visits to be received is highly predictable and the individual plant possess the ability to adjust pollen-dispensing schedules accordingly” by which”plant fitness may increase substantially”^51^. Flower visitation has been shown to be remarkably regular in several species of Loasaceae subfam. Loasoideae for which detailed observations are available^44,45,52^. The floral behaviour reported for this plant group thus complies with the theoretical ideal proposed by LeBuhn and Holsinger^50^ and hence constitutes a prime example to study the evolution of such an elaborate pollen dispensation system.

### Floral function in Loasaceae subfam. Loasoideae

**Figure 1:**
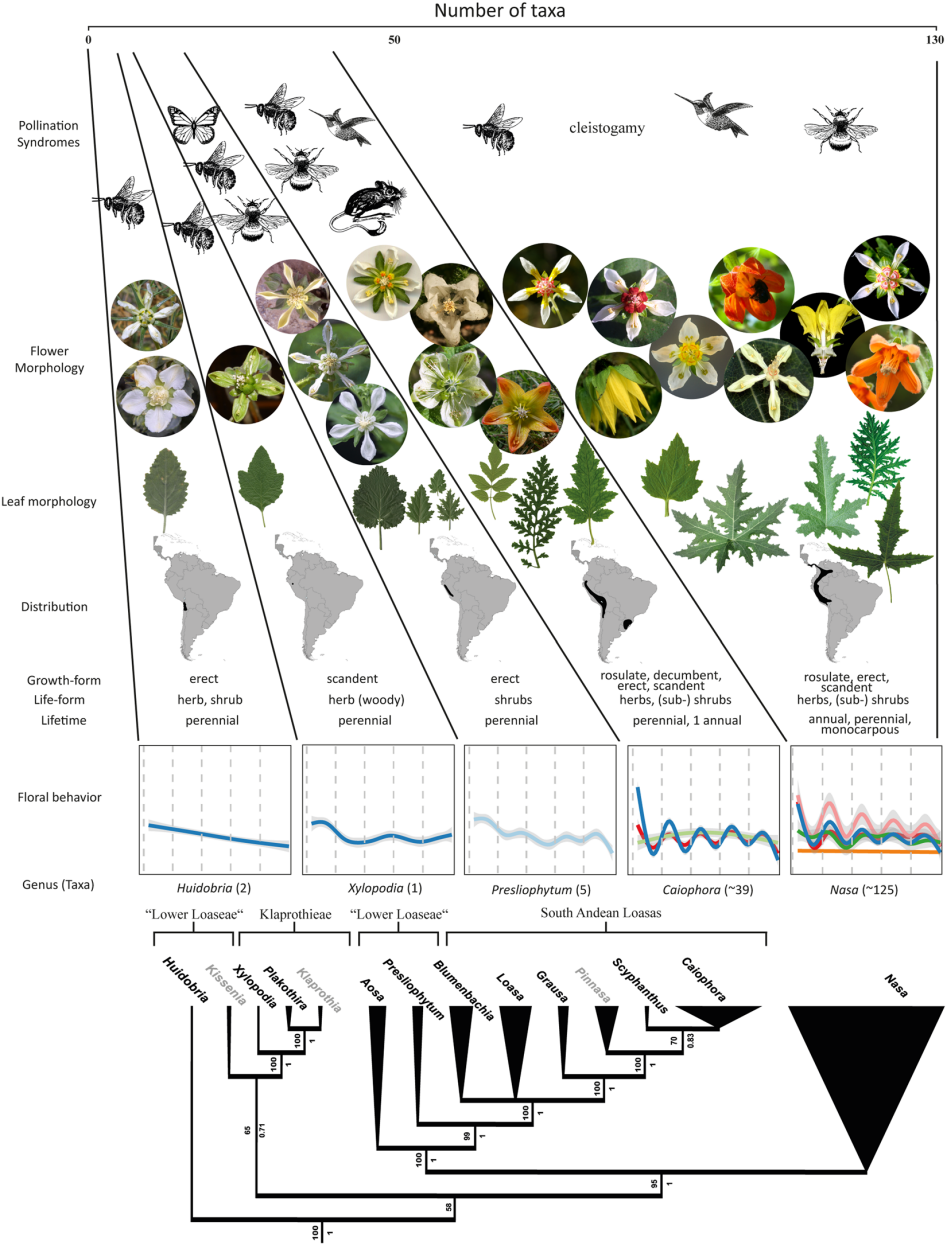
Graphical summary of the diversity found in selected genera of the Loasaceae subfam. Loasoideae. Five genera have been chosen exemplarily to illustrate the general evolutionary trends determined. The tree at the bottom shows the phylogenetic relationship of the whole subfamily with the width of the branches indicating the number of taxa currently accepted (bootstrap values above, posterior probabilities indicated below branches).

**Figure 2:**
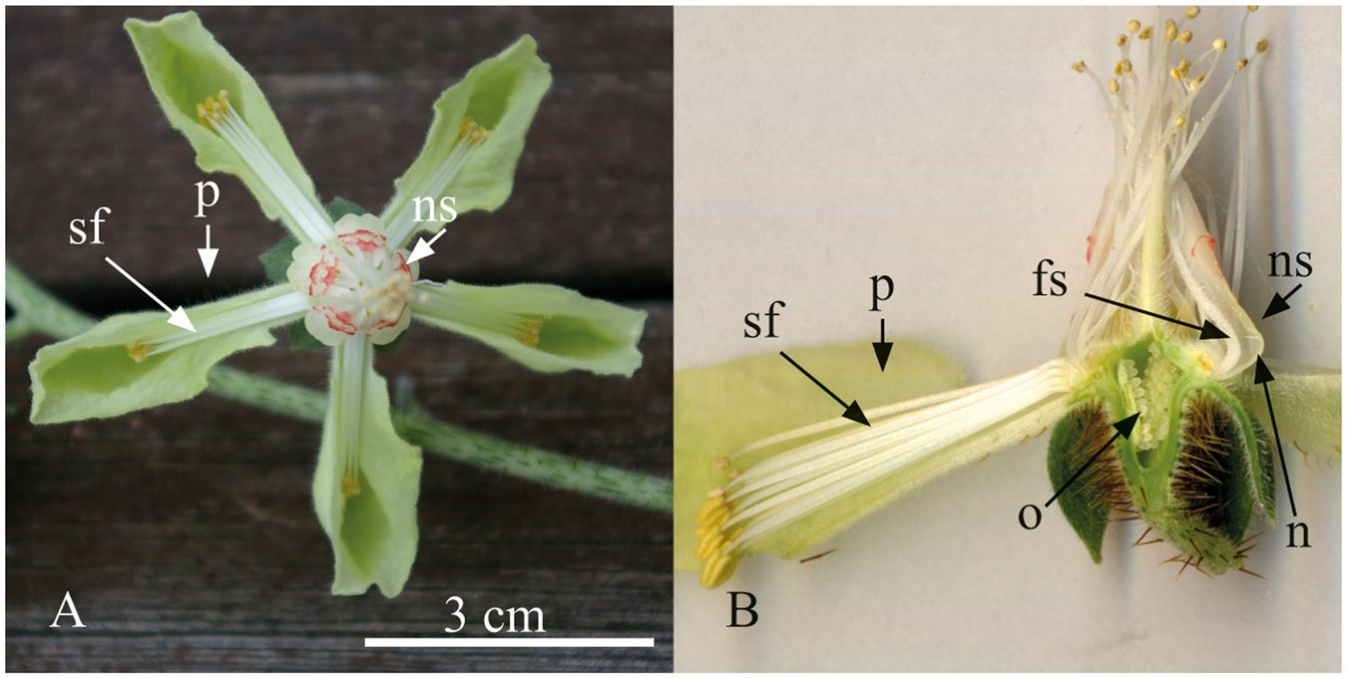
Typical flower of Loasaceae subfam. Loasoideae (*Nasa macrothyrsa*). (**A**) Frontal view, note the stamen fascicles (sf) hidden in the boat shaped petals (p) and the nectar scales (ns) providing a visual cue, structures to hold on and guide the pollinator to the nectar (n). (**B**) Longitudinal section through the flower. The nectar is secreted from the margins of the receptacle (bearing the ovules (o)) into the nectar scales (ns) and accumulates at their base. Two free inner staminodes (fs) direct the pollinator to the nectar.

Loasaceae are a small, predominantly neotropical plant family with a center of diversity in Andean South America (Colombia to Chile). The family comprises ca. 350 species in 21 genera. Molecular studies have largely confirmed earlier systematic re-arrangements based on morphology (e.g.^53^), and the phylogeny of the group can be considered as well-resolved^54–56^. In spite of its relatively moderate number of species, the family is morphologically highly diversified (Fig. 1). Numerous studies have revealed a high level of diversity for growth- and life-forms^57^, leaf morphology and wood anatomy^58–61^, pollen- and seed morphology^62,63^, indumentum^64–66^ and especially floral morphology^67–70^. Most of the floral diversification is found in subfam. Loasoideae comprising ca. two thirds of all species (200 spp.) in 14 genera. Loasoideae are clearly distinguished from the other subfamilies by their deeply boat-shaped petals, into which the immature stamens are initially reflexed, and the highly modified staminodial complex, consisting of an outer, fused floral scale and inner, free staminodia (Fig. 2)^71^. Overall flower morphology is relatively conserved throughout the subfamily, but two tribes are recognized based i.a. on the number of floral organs: tetramerous Klaprothieae (3 genera) and mostly pentamerous Loaseae (11 genera Fig. 1).

The flowers of Loasoideae are polyandrous (many stamens) and show both dichogamy (male and female organs mature at different times) and protandry (stamens/pollen is presented before the stigma becomes receptive), two very common strategies to promote outcrossing in angiosperms^72^. The 10 to 250 stamens are arranged into antepetalous fascicles. They are initially reflexed into the spreading, boat-shaped petals and typically oriented at an angle of 40–140° to the style (Fig. 2). During the staminate phase, stamens mature sequentially and gradually present their pollen^47,67–69^. As the anthers mature, the filaments successively and individually curve at their bases, thus bending the anthers into the center of the flower, where pollen is presented to the flower visitors. The details of the mechanistic principles in the Loasoideae remain unexplained to date but there is evidence that the stamen movement was preceeded by an increased synorganization of the vasculature system in the receptacle, which is known to play a key role in the transduction of electric signals^47^. It can be assumed that the underlying molecular mechanisms to perceive (sense and transmit the stimulus) and respond (stamen movement) to the stimulation of the nectar scale in Loasoideae are the same that are generally recognised for the mechanoperception in plants^37,73,74^.

The movement is fast enough to be observed with the naked eye and typically takes only 1–3 minutes. Throughout the staminate phase autonomous movement takes place during the daylight hours of 2–3 consecutive days, ensuring that fresh, viable pollen for potential pollinators is continuously available in the centre of the flower and pollen offerings in the flower remain more or less constant throughout the staminate phase^46,47^. Additionally, thigmonastic stamen presentation occurs in most genera. Here, the presentation of fresh anthers in the flower centre is triggered by pollinator visits (Supplementary Video). The peculiar staminodial complexes alternate with the filament fascicles and typically consist of five staminodes; the outer three of which are fused into a scale-shaped structure (floral scale) and two of those close this scale towards the center of the flower (Fig. 2). These staminodial complexes have a range of different roles in plant-pollinator interaction (for details see^69,70^). Nectar is secreted from the margins of the receptacle into the floral scales, with the nectar continuously replenished^45^. To access the nectar, pollinators insert their proboscis or beak into the floral scale forcing it to bend outwards. This manipulation of the floral scale triggers the thigmonastic movement of filaments^44,75^. Unlike the autonomous movement, this thigmonastic motion is a direct reaction to a floral visit and thus plant behaviour that is active and responsive^45–47^. Unlike autonomous stamen presentation, thigmonastic stamen presentation replenishes the pollen offerings of the flower immediately after a pollinator visit. Therefore, the time period where the flower is not able to dispense pollen to a flower visitor is reduced. This complex floral behaviour has been demonstrated for a range of species from different genera (*Blumenbachia*, *Caiophora*, *Loasa*, *Nasa*, *Presliophytum*) in Loasaceae subfam. Loasoideae^45–47,52,76,77^, and has not yet been reported from representatives outside this subfamily that lack both reflexed stamens and floral scales. Comparative data have not been provided on floral responses across different taxa, nor has an evolutionary assessment been attempted. To the best of our knowledge the present study is the first attempt to explain plant behaviour – in our case a highly specific, thigmonastic response to flower visits – in a phylogenetic context across many (in our case 44) species representing circa ¾ of all genera (11 out of 14) of the subfamily.

### Aims

Based on what is known, the floral function of Loasaceae represents a unique system for an evolutionary study on plant behaviour, in this case the specific reaction of stamens to pollinator visits. The present paper presents a large experimental data set on the behavioural diversity of thigmonastic stamen presentation and places it in the context of a phylogenetic framework of a molecular phylogeny and data on the pollination syndromes of neotropical Loasaceae subfam. Loasoideae. Based on these data we aim at:

1. Investigating the presence and characteristics of the stamen presentation across the subfamily.
2. Documenting the extent of diversification of thigmonastic stamen presentation as plant behaviour.
3. Contextualizing the patterns of thigmonasty with the phylogeny of the group and the pollination syndromes that have been documented.
4. Assessing and discussing thigmonasty as a behavioural expression and investigating its possible significance for the evolutionary history and diversification of this plant group in the overall context of flower function.

## Material and Methods

### Plant material

A total of 44 taxa from 11 genera of Loasaceae subfam. Loasoideae were investigated (*Aosa* (2 species), *Blumenbachia*^3^, *Caiophora*^12^, *Grausa*^1^, *Huidobria*^1^, *Loasa*^5^, *Nasa* (13 species and subspecies), *Plakothira*^1^, *Presliophytum*^2^, *Scyphanthus*^2^ and *Xylopodia*^1^, for a complete list incl. taxonomic information see Supplementary Table 1). The data for *Huidobria fruticosa* were obtained from plants in their natural habitat. All other datasets were obtained from plants in cultivation. All species were raised from seed collected in the wild, with the only exception of *Blumenbachia insignis* and *B*. *hieronymi*, which were obtained from cultivated material of unknown provenance from botanical gardens (see Supplementary Table 1 for detailed voucher information). Plants were cultivated in the greenhouses at the Institut für Biologie, Freie Universität Berlin (2001 to 2008) and the Nees Institut für Biodiversität der Pflanzen, Universität Bonn (2012). For detailed information on cultivation see^41^.

### Pollinator Data

Pollinator data for the taxa studied were either extracted from the literature or are based on our own field observations. For some taxa the pollination syndrome were extrapolated from the overall flower morphology and data available on closely related taxa. Pollination syndromes are generalized to the principal types observed in the Loasoideae^70,75–78^. Six different groups of pollinators have been previously reported for Loasoideae: short-tongued bees, long tongued bees, flies, butterflies, hummingbirds and mammals (Supplementary Table 2). Based on field observations, observations in cultivation and literature data the taxa examined were assigned to eight different pollination syndromes for the present study: short-tongued bees, long tongued bees, long tongued bees and hummingbirds, flies, various insects (i.a. butterflies), hummingbirds, mammals and cleistogamy.

### Thigmonastic stamen movement

Depending on the quantity of flowers available, experiments were either conducted with isolated inflorescence branches placed into glass vials in the laboratory or were carried out directly on living plants in the greenhouse. Flowers were individually marked and mature stamens that already had moved into the center of the flower were cut off one hour prior to the first stimulation experiment. Depending on flower availability, 10–35 flowers were used for individual sets of experimental observations with control groups of 5–22 flowers. Stamen movement was triggered by imitating a pollinator visit by slightly bending all five nectar scales outwards with a needle. Anthers of the newly moved stamens were carefully cut off to preclude double counting. Five consecutive stimuli with 30 minute intervals between the individual stimuli were carried out. This stimulus interval was chosen based on field observations indicating an average interval between two visits to individual flowers of ca. 25 minutes for one of the species^45^. This follows the rationale that the timing of experimental visits to flowers should reflect the natural visitation rate^51^ and at the same time serves the purpose to standardize the resulting dataset. For purposes of recording, the overall interval of 30 minutes was subdivided into fractions of 5 minutes each and the anthers moved in each of these 5 minute sub-intervals were pooled to ease data capture and analyses, resulting in a rate of stamens moved per 5 minute intervals.

### Statistical analysis

In order to test for the presence of thigmonastic stamen movement in the species investigated, we applied multiple Generalized Additive Models (GAMs), as implemented in the gam() function of the mgcv package^79^ in the R framework^80^. We used one smoother per treatment (control vs. stimulation) and the treatment as categorical variable to predict the average number of moved stamens per 5 minute interval after the manual impulse. To account for false discovery rate due to multiple comparisons, we adjusted p-values using the Benjamini-Hochberg procedure^81^. Details of data exploration procedure and modeling terms can be found in Supplementary Material 3.

For the comparison of thigmonastic patterns between pollination syndromes across phylogenetic placements, we applied a global Generalized Additive Mixed Model (GAMM), as implemented in the function gamm() in the mgcv package, to the whole dataset from which control flowers were removed. Details on model term selection and model validation can be found in Supplementary Material 3. In brief, we predicted the number of moved stamen per 5 minute interval by the respective impulse and the pollination syndrome. To account for phylogenetic relatedness of sampled species, we included the distance to the root as calculated with the function distRoot() in the adephylo package^82^ for the phylogenetic tree as described below. Since we performed multiple stimuli at single flowers, which are not independent of each other, we included the impulse period into the modeling term and included the taxon ID as random factor.

To test if phylogenetic radiation impacts the thigmonastic stamen presentation in flowers pollinated by short-tongued bees, we removed control treatments and other pollination syndromes from the dataset and calculated a separate GAMM. We predicted the number of moved stamens per impulse period, with the impulse, the absolute experimental time, and the genus ID ordered according to phylogenetic placement. We added a correlation structure for the impulse period, and the species ID as random factor. Model selection and validation can be found in detail in Supplementary Material 3.

### Molecular methods

The taxon sampling for the molecular data conforms exactly to that of the pollination data and thigmonastic stamen movement. Whenever it was possible, we attempted to use the same voucher specimens for the experimental as well as the molecular data. Some taxa were represented by more than one accession (*Nasa moroensis*, *N*. *olmosiana* and *N*. *triphylla* subsp. *triphylla*) if the taxa were morphologically variable. Additionally *Gronovia scandens*, *Mentzelia albescens*, *Eucnide urens* and *Deutzia discolor* were included in the analyses as outgroups. Outgroups were selected based on the phylogenetic studies of Weigend *et al*.^54^ and Hufford *et al*.^55^. All sampled plant material with its geographic origin, herbarium voucher, and GenBank accession numbers is listed in Supplementary Table 1.

DNA was extracted from 0.5–1 cm^2^ samples of silica gel dried leaves or herbarium leaf material with a modified CTAB method^83^. We sequenced the plastid regions *trn*L*-trn*F, *mat*K, the *trn*S*-trn*G intergenic spacers, and the *rps*16 intron (taxon sampling was complete for all markers). The PCR amplification and sequencing protocols follow Acuña *et al*.^56^. Sequences were assembled in Geneious v. 8.0.1^84^ using the default *De Novo* assemble settings.

Assembled sequences were aligned in Mafft v. 7^85^, followed by manual adjustments using PhyDE v. 0.9971^86^. Alignment files are available from the corresponding authors on request. FindModel (available from http://hcv.lanl.gov/content/sequence/findmodel/findmodel.html), which implements Posada & Crandall’s^87^ Modeltest, selected GTR+Gamma as the model that best fits all four plastid markers. Phylogenetic reconstructions for Maximum Likelihood (ML)^88^, were conducted in RAxML v. 8^89^ included in RAxMLGUI v. 1.5 Beta^90^. Bayesian Inference (BI)^91^ was conducted in MrBayes 3.2.2^92^, in the CIPRES Science Gateway computing facility^93^. Each marker was at first analyzed separately. In the absence of topological conflict (defined as incongruence in the topologies of nodes with bootstrap support >80%) the markers were combined. ML analyses were implemented using the GTRCAT approximation, because it works in an analogous way to GTR+Gamma and yields similar results but with less intensive computational costs^89^. The statistical support for the nodes was assessed by 1000 ML thorough bootstrap replicates with 100 runs under the same analysis conditions. The BI was conducted, with four independent runs with one cold and three heated chains, the Markov chain had a length of 10 million generations, sampled every 1000 generations. After convergence was assessed in Tracer 1.5^94^, the first 2.5 million generations were discarded as burn-in.

### Phylogenetic effects

Traits of any kind are usually more similar between closely related species than between more distantly related species and therefore, they cannot be regarded as independent samples^95^. Therefore, it is necessary to account for phylogenetic distance in any analysis of attributes across related species^96^. Comparative phylogenetic methods have been used to investigate whether traits of species are influenced by their ancestral state^95,97,98^. Testing for phylogenetic signal thus permits an evaluation of whether phenotypic differentiation of a given species trait is equal to, higher than or less than what would be expected under a Brownian motion (BM) model of evolution^97,98^. A given trait can be treated as independent of phylogenetic history if there is no significant phylogenetic signal^96^.

To investigate whether variation of thigmonastic stamen movement between species is influenced by phylogenetic history, we calculated Blomberg’s K^98^ and Pagel’s λ^97^ and tested these values for significance. As continuous variables of thigmonastic stamen movement, we used the average number of stamens moved within the first 5 minutes and the average number of stamens moved after 30 minutes (note that the average number of stamens moved after 30 minutes corresponds the below mentioned **s**tamen movement per stimulus per flower in the 30 Min-interval following an individual **s**timulus (sps30 hereinafter)). As a measure of stamen movement speed we calculated the percentage of stamens which moved within the first 5 minutes relative to the stamens moved after 30 minutes. These variables were tested for phylogenetic signal for the stimulation treatment as well as for the control treatment. This results in six variables which were tested for a significant phylogenetic signal: four variables of stamen movement and two of stamen movement speed.

The underlying branch lengths were based on the rooted maximum likelihood phylogenetic reconstruction computed with RAxML v. 8^89^ as described in the previous section. Branch lengths of the trees are proportional to the substitution rates per site and so the distance to the root will differ for the different tips. Smith & Donoghue^99^ and Lanfear *et al*.^100^ have shown that rates of molecular evolution in plants could change according to life history and growth form. Accordingly, we assume that molecular markers can have variable evolutionary rates.

Prior to testing for phylogenetic signal, the outgroups used for constructing the phylogeny were trimmed from the tree with the drop.tip() function in the R package ape^101^. Blomberg’s K and Pagel’s λ were calculated with the phylosig() function implemented in the R package phytools^102^. For testing if the observed K value is significant we applied a randomization test implemented in the phylosig() function based on 10000 randomizations of the trait datasets to generate a null distribution. For testing the significance of λ a likelihood ratio test implemented in the phylosig() function was applied. This test indicates whether the reported λ significantly differs from a λ equal to zero (i.e., a “star phylogeny”) where relatedness does not explain the trait similarity between species.

## Results

### Thigmonasty

38 of the 44 taxa examined show a significant thigmonastic response upon a stimulation of the nectar scales in terms of significant differences in the rate of stamen movements compared to control flowers (Supplementary Material 3: Fig. 8). The remaining six taxa show autonomous stamen movement only, a thigmonastic response is absent. Of these taxa, *Huidobria fruticosa* and *Xylopodia klaprothioides* belong to the early-branching grade of subfam. Loasoideae. *Presliophytum incanum* and *Aosa rupestris* are part of the speciose Higher Loaseae-clade of the Loasoideae. For *A*. *rupestris*, stamen presentation has already been reported to only be autonomous^103^. Interestingly, stamen presentation in respective sister taxa of these non-thigmonastic species is thigmonastic (*P*. *heucheraefolium* and *A*. *parviflora*). The other non-thigmonastic species include mammal-pollinated *Caiophora coronata*^77^ and cleistogamous *Nasa chenopodiifolia* (pers. observation). All other taxa examined show a significant reaction upon scale manipulation mimicking pollinator-behaviour and are known to be pollinated by insects and/or hummingbirds (Supplemental Material 3: Fig. 8).

The control groups show random, aperiodic stamen presentation. Theoretically, the autonomous stamen movement should approach a straight line if sample size was large enough and observation period time long enough. Due to the very low overall autonomous stamen presentation rate, single movements have a strong influence on the shape of the curve in our analyses. The resulting shapes (Supplementary Material 3: Fig. S8) thus mostly represent random patterns rather than straight lines.

**Figure 3:**
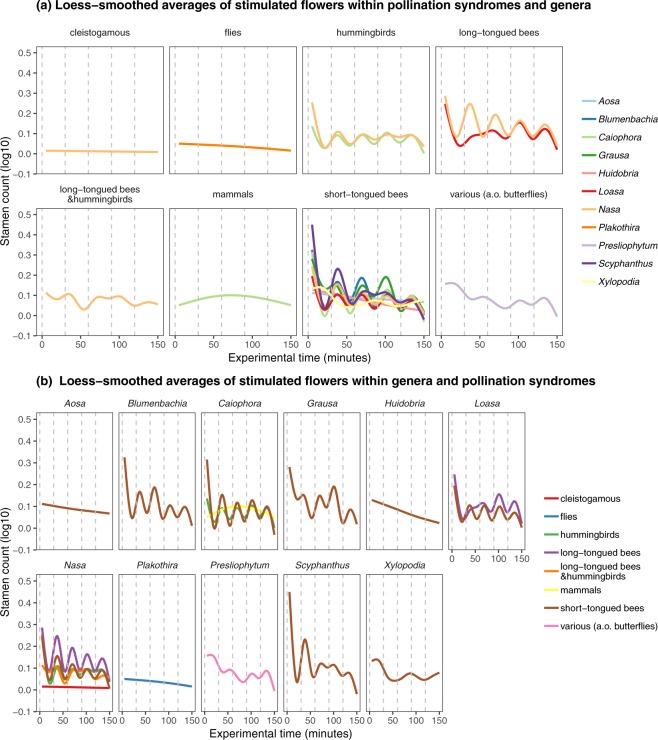
Differences in staminal movements during experimental time in reaction to manual stimulations of the floral organs in flowers. Dashed vertical lines mark stimulations. First evaluation of staminal reaction occured 5 minutes after stimulus. Solid lines are averaged Loess-smooths colored for each pollination syndrome (**a**) or genus (**b**). Shaded ribbons show 95% confidence intervals of smooths.

On average, a total of 0.24–4.24 stamens per flower move in the 30 Min-interval following an individual stimulus (sps30). The thigmonastic reaction is weakest in *Nasa chenopodiifolia* (sps30 = 0.24) and highest in *Scyphanthus stenocarpus* (sps30 = 4.24). A comparison of different taxa reveals considerable differences between the genera. Whilst all the species of *Caiophora* show a rather uniform presentation rate of 1.22 to 2.94 sps30, *Nasa* displays a more variable response of 0.24 to 3.39 sps30. Even small genera such as *Presliophytum* and *Scyphanthus* exhibit striking differences between individual taxa with 0.8–2.67 and 2.19–4.24 sps30, respectively. There is no obvious, quantifiable trend in the movement rates either across the whole subfamily or within genera (Fig. 3a). Comparing patterns within pollination syndromes rather than taxa, the movement rate also varies strongly. In hummingbird pollinated taxa, for example, 0.76 to 3.28 sps30 are recorded, in taxa pollinated by short-tongued bees stamen presentation rates vary from 0.66 to 4.24 sps30. The other insect and the rodent pollinated taxa also fall into this range; the only exception is the cleistogamous *N*. *chenopodiifolia*, displaying the lowest movement of all taxa examined (0.24 sps30).

**Figure 4:**
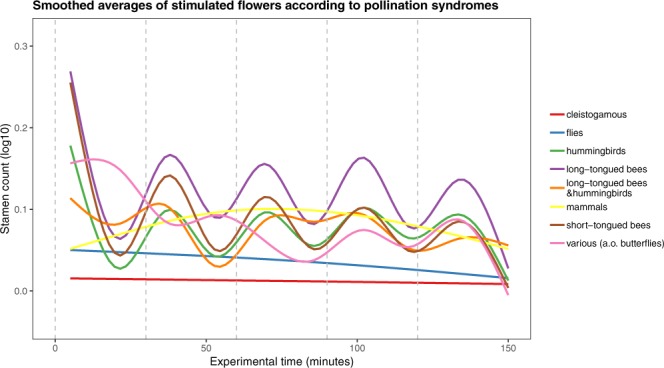
Differences in staminal movements between pollination syndromes during experimental time in reaction to manual stimulations of the floral organs. Dashed vertical lines mark stimulations. First evaluation of staminal reaction occured 5 minutes after stimulus. Solid lines are estimated Loess-smooths colored for each group of pollination syndrome, including species across all genera. Shaded ribbons show 95% confidence intervals of smooths.

Figure 3 shows the average stamen movements over time, pooled for the different genera sorted by pollination syndromes (Fig. 3a), respectively pooled for the pollination syndromes and sorted by genera (Fig. 3b). Taxa pollinated by bees and hummingbirds show an overall rhythmic stamen presentation, synchronised by the repeated stimuli. The other syndromes are characterised by an asynchronous presentation pattern (mammals, other insects and mixed pollination), or show no dynamics in the movement at all (cleistogamy). Within the common syndromes in Loasoideae, i.e. bee or hummingbird pollination, a strong variation can be found across the taxa examined. Variation is highest in bee pollination and lowest in hummingbird flowers. Figure 4 summarizes the overall patterns observed for the different pollination syndromes. It reveals rhythmic patterns that are more or less synchronous to the stimuli for all taxa

**Figure 5:**
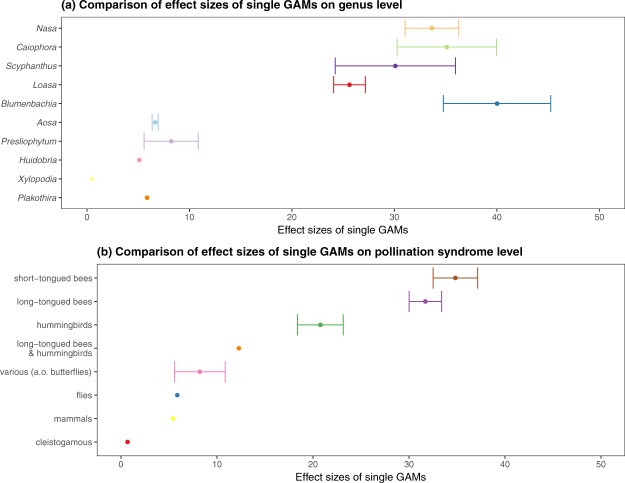
Comparison of GAM effect sizes averaged for different genera (**a**) or pollination synsdromes (**b**) comparing staminal response after experimental stimuli to non-stimulated control flowers within each species. Points show mean effect sizes per group. Bars refer to standard errors. Genera (**a**) are ordered ascendingly to increasing distance to the node in phylogenetic tree. Pollination syndromes are ordered to increasing average effect size.

Stamen presentation patterns are mainly influenced by pollination syndrome and to a lesser extent by the phylogenetic distance between the taxa (Fig. 5). Comparing the effect size of single GAMs on the pollination syndrome and the genus level, the standard error is smaller (and remarkably uniform) throughout the different pollination syndromes examined. In order to understand the adaptation of individual taxa to a specific pollination syndrome during the evolutionary history of the group, we analyzed a reduced dataset of all taxa pollinated by short-tongued bees only. It has been argued that this pollination mode constitutes the plesiomorphic condition in Loasoideae^53,70,78^ and it is universally found in eight of the eleven genera examined, including species-poor *Xylopodia* and *Huidobria* and species-rich *Nasa* and *Caiophora*.)

Representatives from the basal nodes (*Huidobria* and *Xylopodia*) show decreasing, *Aosa* rather random reactions upon repeated stimulation. Figure 3b illustrates that there is an increase in the regularity of the reactions in the other clades (e.g., *Nasa*, *Caiophora* and *Blumenbachia*), in line with the analyses of the effect sizes across all datasets. Furthermore, these taxa maintain a virtually standardized response over repeated flower visits, presenting uniform stamen numbers with largely uniform timing.

### Phylogenetic signal in stamen presentation

The phylogenetic placement of individual taxa, included as distance of branch tips to the root (of the phylogenetic tree) in the final GAMM, has only a marginal effect (F = 0.197, p = 0.657) on thigmonastic patterns (Supplemental Material 3). However, the effect size of single pairwise GAMs – that is the difference in shape between thigmonastic and autonomous movement – increases with increasing number of branches between the respective clade and the common node (Fig. 5a). Testing the phylogenetic signal with Blomberg’s K for the average stamen movement after 5 minutes and after 30 minutes as well as for the speed of the stamen movement revealed no significant K value (Table 1). As a more robust approach Pagel’s λ only revealed a λ of 0.636 (i.e., significantly different from zero p = 0.0007) for the speed of stamen movement in the stimulation treatment. The consistent non-significance of the very low K and of the λ values for stimulated stamen movement (i. e., thigmonastic movement) indicates that if there is an effect of shared ancestry, it is very weak (Table 1).

**Table 1:** Phylogenetic signal and statistical tests for variables of stamen movement in the Loasoideae.

		Blomberg’s K	Pagel’s λ
Movement	Stamen movement 1^st^ 5 min (stimulation)	0.087	0.383
	Stamen movement 1^st^ 5 min (control)	0.100	0.154
	Stamen movement in 30 min (stimulation)	0.096	0.000
	Stamen movement in 30 min (control)	0.165	0.122
Speed	% of stamens moved in 1^st^ 5 min (stimulation)	0.091	0.636*
	% of stamens moved in 1^st^ 5 min (control)	0.089	0.077

The asterisk indicates significance at the 95% confidence level based on a randomization test for Blomberg’s K and on a likelihood ratio test for Pagel’s λ.

## Discussion

### Thigmonastic patterns

**Figure 6:**
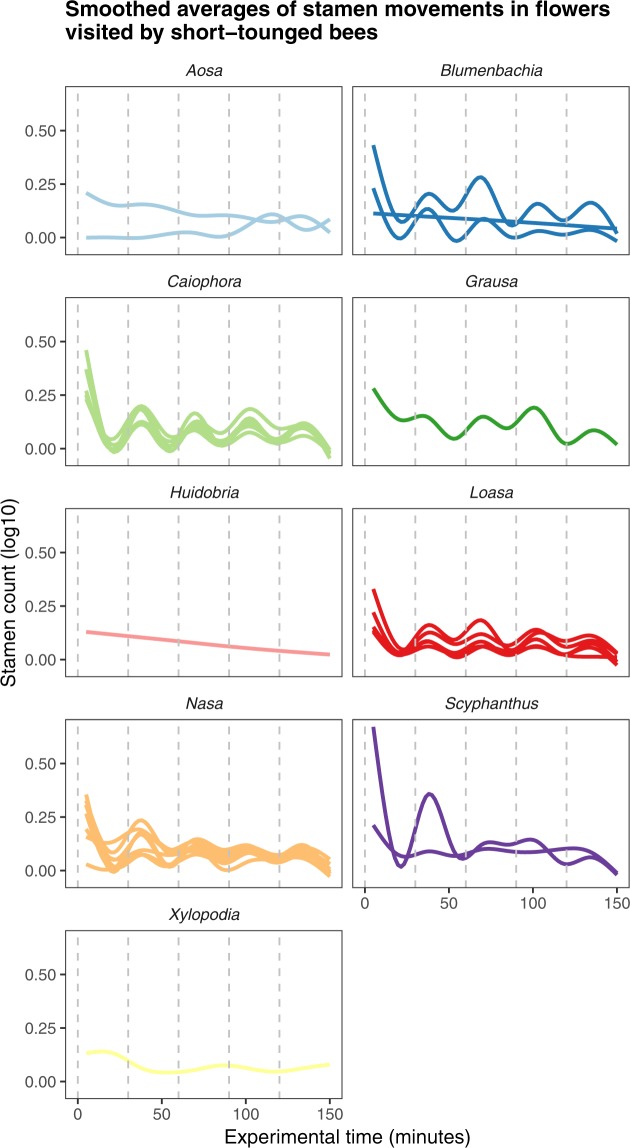
Differences in staminal movements between and within genera during experimental time in reaction to manual stimulations of the floral organs in flowers pollinated by short-tounged bees. Dashed horizontal lines mark stimulations. First evaluation of staminal reaction occured 5 minutes after stimulus. Solid lines are estimated Loess-smooths, colored for each genus.

*Huidobria*, *Plakothira* and *Xylopodia* only show an autonomous movement (Fig. 1). Some taxa of the basal grade lack floral scales (*Klaprothia mentzeliodes*) and/or are obligate selfers (*K*. *fasciculata*). Overall, it can be assumed that these early diverging lineages of Loasoideae indeed show autonomous pollen presentation only and that this represents the ancestral condition, although experimental evidence on the two other basally branching taxa (*H*. *chilensis*, *Kissenia*) would clearly be desirable to corroborate this conclusion. The vast majority of taxa investigated display thigmonastic stamen presentation. A thigmonastic response can be triggered – often with highly predictable timing – by mimicking a pollinator visit by manipulating the floral scale. The analyses further indicate that – in very general terms – the thigmonastic stamen movement increases with increasing distance from the phylogenetic root in effect size, speed, and regularity. This appears to reflect an increasing ability to control and adjust pollen presentation to a given flower visitation scenario. Basally branching taxa show simple, if any thigmonastic stamen presentation and do not fall into a rhythmical pattern of pollen presentation in reaction to periodic re-visits. In representatives of the terminal clades of the Loasoideae, movement patterns are highly predictable and are synchronized with repeated flower visits (Figs 1, 3b and 6). Minor adjustments of the thigmonastic pollen presentation indicate either an adaptation to whole pollination syndromes, or possibly to the idiosyncratic visitation behaviour of individual pollinator species (Fig. 3a). Flowers pollinated by short-tongued bees - the largest subset of the taxa here studied - show a remarkably homogeneous thigmonastic pattern across the genera (Fig. 6). Minor deviations from this relatively uniform floral reaction may be the result of random effects of factors such as flower size and morphology or may go back to fine-tuning in response to different behavioural patterns in this diversely pollinated group. Long-tongued bees and hummingbirds have a larger body surface and are capable of carrying larger pollen loads compared to short-tongued bees. The presentation of a high number of stamens presented may consequently be advantageous for plants pollinated by these larger animals (Fig. 3a). However, additional datasets indicate that pollen load might be adjusted at least partly by increasing anther size and pollen grain number (Henning & Weigend, in prep.) rather than by shifts in the thigmonastic response, i.e. the number of stamens presented. Increasing the rate of anther presentation would automatically diminish the scope for pollen partitioning, since the anther stock would be depleted much faster. It is also obvious that hummingbird-pollinated taxa possess a reduced thigmonastic response to the second stimulus (Fig. 3a), which likely corresponds to specific pollinator behaviour. Hummingbirds are known to be erratic trapliners, foraging over long distances and returning after long and irregular time intervals^104,105^, rendering iterative pollen replenishment in short intervals ineffective. The behavioural differences between plant taxa that are visited by different pollinator groups therefore appear to reflect the differential interaction with different pollinators and/or pollinator guilds. Conversely, a secondary loss of thigmonasty can be inferred for *Caiophora coronata*, *Nasa chenopodiifolia*, and possibly *Presliophytum incanum* and *Aosa rupestris*. *Caiophora coronata* is reportedly pollinated by opportunistic rodents whose visitation rate may be highly randomised and possibly with one off visits to individual flowers^77^. *N*. *chenopodiifolia* is largely autogamous or even cleistogamous – any form of pollen partitioning and timing of pollen presentation would therefore be superfluous. In the case of *Presliophytum* and *Aosa*, thigmonasty is significantly different from experimental controls in only one of two closely related taxa studied (Fig. 8 in Supplementary Material 3). Additional studies on other species of *Aosa* would clearly be of interest, but observations of cultivated individuals indicate that species of *Aosa* cultivated so far are highly autogamous, possibly relaxing the need for fine-tuning pollen presentation to pollinators. *Presliophytum incanum* could be shown to have a very broad range of flower visitors from different insect groups, with butterflies representing a considerable proportion of the observed pollinators, and our data show that it does not show a thigmonastic response. Conversely, for *P*. *heucheraefolium* only a narrow range of visitors has been reported, essentially long-tongued-bees, and it does show a thigmonastic response (*Presliophytum* sp., Fig. 8 in Supplementary Material 3). This would underscore that a thigmonastic response only makes adaptive sense when the range of pollinators is narrow and predictable in its behaviour.

### Phylogenetic signal

Patterns of thigmonastic stamen presentation in the plants investigated in the present study indicate an adaptation to pollinator groups rather than a correspondence to phylogenetic placement. As indicators for a more controlled and accurate reaction, we present both the effect size of single GAMs (Fig. 5a) and the speed of stamen movement (Table 1), both of which increase in more speciose clades such as *Blumenbachia*, *Nasa* and *Caiophora* and the latter being the only behavioural trait for which we detected a significant phylogenetic signal, based on Pagel’s λ (Table 1). In other words, stamen presentation patterns in distantly related taxa with the same pollination syndromes are more similar than those of closely related taxa with different pollination syndromes.

Furthermore, effect size is positively correlated with the phylogenetic “derived-ness” (Fig. 5a), i.e., the complexity and the intensity of the reaction upon a stimulus increases with the increasing distance from the root of the phylogenetic tree. An increasing precision of the thigmonastic response can also be detected when looking more closely at the average responses of short-tongued-bee pollinated taxa upon individual stimuli. Within the derived genera, such as *Nasa* and *Caiophora*, precision of the response increases towards the crown group, specifically the speed of the thigmonastic response shows a continuous increase. The thigmonastic patterns in flowers visited by short-tongued bees are relatively stable within individual genera, whereas the regularity of these patterns (smooths) seems to increase in the more derived genera.

Floral adaptations to functional pollinator groups have been shown to be closely associated with speciation events^106,107^, and our data indicate that this might be also the case in the Loasoideae. Adaptations of floral traits are at the heart of reproductive isolation and have been shown to be subject to significant phylogenetic signal (e.g.^108,109^). The lack of phylogenetic signal for stimulated stamen presentation suggests that the evolutionary adjustment of thigmonastic stamen presentation in Loasoideae is relatively rapid and possibly a *de novo* invention. An absence of a phylogenetic effect has been suggested to either arise through rapid evolution and multiple homoplastic transitions^110^ or could be explained by a high degree of adaptability in behavioural responses. Previously, it has been argued that Loasoideae species show a fast evolutionary adjustment of nectar amount and composition with shifts in pollination syndrome^78^. It is possible that the rapid adjustment of pollen presentation schedules is a complementary mechanism to the evolution of nectar characteristics in response to pollinator shifts.

### Floral behaviour and speciation

It has been argued that “…much plant taxonomy relies on flower structure in which plasticity is minimized” Trewavas (p. 15^111^) It is undoubtedly true in general terms that the basic architecture of Loasoideae-flowers is remarkably conserved^112^. This argument could be contrasted with the notable behavioural diversity documented here for the first time, but this would underestimate the extreme diversification in the details of flower morphology (Fig. 1), in regards to aspects of function and signalling^60,62,63,70^. Similarly, the primary floral reward in Loasoideae is highly diverse and the broad range of nectar amounts and concentrations has been shown to correlate with pollination syndromes^78^. Consequently, the adjustment of flower behaviour, i.e. the amount, timing and periodicity of pollen presentation in reaction to flower visits, appears to be part of a complex evolution of floral function in tandem with aspects of signal, reward, and morphology. This functional complexity permits multidimensional adaptations to specific individual pollinators or pollinator groups. The high level of diversity and the elevated rate of micro-endemism characteristic of this plant group has been attributed to temporal habitat heterogeneity (e.g., landslides) and repeated re-colonization of Andean habitats, in particular by the annual species (e.g., *Nasa*^113^) In order to ensure the rapid establishment of stable populations after a successful initial colonization of a new habitat, reliable pollen vectors are vital. It has been argued that an increasing adaptation of a plant taxon to a specialized pollinator following its initial recruitment is often followed by a stepwise consolidation of a mutualistic relationship^114^, in turn giving rise to pre-mating barriers to the parental population. In Loasoideae, this includes a specific floral signal, morphology and reward (amount and concentration of nectar) and a – possibly rapid – adjustment of the pollen presentation timing to specific pollinators and their idiosyncratic visitation rates. We hypothesize that thigmonastic stamen presentation is a mechanism to increase male fitness^45^ and has been one important component in the diversification of Loasoideae in Andean habitats, further strengthening the divergence of populations by adding an additional dimension to potential pre-mating barriers between diverging plant populations. The variation in chromosome number seems an important driver of the diversification of *Mentzelia* (Loasaceae subfam. Mentzelioideae^115,116^) where aneuploidy and polyploidy act as reproductive isolators. There is no evidence for this in Loasoideae, with usually highly conserved karyotypes^53,117,118^. Thigmonastic pollen presentation with characteristic – and apparently evolutionarily labile – timing should be considered in concert with complex adjustments of floral signal, nectar quality and quantity, flower orientation and functional morphology (nectar scales), providing numerous opportunities for adaptation and specialization along multiple functional axes.

We conclude that active floral behaviour may be an underestimated component of flower function. A critical review of other traits such as floral scents and stigmatic reactions or even systemic responses to changing pollination scenarios or flower symmetry on the inflorescence- or individual flower-level would likely provide crucial insights into hitherto overlooked mechanisms of plant adaptation and diversification. Clearly, Loasoideae provides an extreme example due to the complexity, speed and precision of floral responses to pollinator induced stimuli. However, the current pattern of plant behaviour and related floral phenomena suggest that flowers could adjust to pollinator preferences and that this ability might convey competitive evolutionary advantages. It is conceivable that many other evolutionary similarly labile behavioural traits related to plant mating exist but have not been recognized due to their low speed or due to the absence of movement. Dynamic nectar replenishment might be a similar, but subtler behavioural response to preferences and visitation rates of individual pollinators^119,120^. An exemplary survey indicates that such a response is likely common throughout flowering plants^121^ and a recent study discusses the characteristics of nectar secretion dynamics in the context of mixed pollination syndromes^122^. Irrespective of future insights, here we demonstrate that Loasoideae flowers show a rather sophisticated behaviour and we are able to provide a general outline of the evolutionary pathways of complex thigmonastic responses. This is the first time such an evolutionary scenario is proposed for plant behaviour. It invites a plethora of further studies, e.g. on the details of plant-pollinator relationships, but more importantly on the physiological details of mechanoreception in plants, the anatomy and physiology of the transmission of the stimulus and the basis of the mechanical response specifically in Loasoideae flowers and for plants in general. Finally, it is evidently time to investigate the genetic basis for plant behaviour – since we demonstrate here that it is a trait, that evolves and diversifies like any other morphological or chemical trait.

## Electronic supplementary material

Stamen movement video

Supplementary Table 1

Supplementary Table 2

Supplementary Material 3

## Data Availability

The datasets generated during and/or analysed in the current study are available in the [Open Science Framework] repository, [https://osf.io/sd4q9/?view_only=5e9563caee50457d851d16efd7b1440d].
