# Supplementary material for: A case of behavioural diversification in male floral function – the evolution of thigmonastic pollen presentation
Source: Sci Rep. 2018 Sep 19;8:14018. doi: 10.1038/s41598-018-32384-4 (PMC6145904; doi:10.1038/s41598-018-32384-4)
Supplement: Supplementary file 2 — Supplementary Table 1 [file 41598_2018_32384_MOESM2_ESM.pdf]

A case of behavioral diversification in male floral function – the evolution of thigmonastic pollen presentation

Supplementary Table 1
Plant material

Tilo Henning, Moritz Mittelbach, Sascha A. Ismail, Rafael H. Acuña-Castillo, Maximilian Weigend

Taxon	Voucher: Floral data	Lab Code	Voucher: Molecular data	GenBank codes			
				trnL-trnF	matK	trnS-trnG	rps16
Aosa parviflora	Grant 4650 (BSB)	W4000	Grant 4650 (BSB)	KY286967	KY286698	KY286877	KY286787
Aosa rupestris	Weigend 7138 (BSB)	W1289	Weigend 7138 (BSB)	KY286925	KY286657	KY286835	KY286745
Blumenbachia hieronymi	Ackermann 601 (BSB)	W2846	Ackermann 601 (BSB)	KY286966	KY286697	KY286876	KY286786
Blumenbachia insignis	Weigend 7475 (B)	W1622	Weigend 7475 (B)	KY286964	KY286695	KY286874	KY286784
Blumenbachia latifolia	Weigend 9135 (B)	W1383	Schwabe s.n. (1958) (B)	KY286949	KY286680	KY286859	KY286769
Caiophora andina	Schlumpberger 663 (LPB)	W4520	Moreira & Luebert 2379 (SGO)	KY287005	KY286735	KY286915	KY286825
Caiophora arechavalatae	Weigend 9330 (BONN)	W4003	Weigend 9330 (BONN)	KY286970	KY286701	KY286880	KY286790
Caiophora canarinoides	Ackermann 395 (BSB)	W4011	Ackermann 375 (BSB)	KY286975	KY286706	KY286885	KY286795
Caiophora carduifolia	Ackermann & Kollehn 288 (BSB)	W1343	Ackermann & Kollehn 288 (BSB)	KY286939	KY286671	KY286849	KY286759
Caiophora cernua	Ackermann 1100 (BONN)	W4007	Ackermann 1100 (BONN)	KY286972	KY286703	KY286882	KY286792
Caiophora chuquitensis	Ackermann 1101 (BONN)	W4022	Ackermann 1101 (BONN)	KY286983	KY286714	KY286893	KY286803
Caiophora cirsiifolia	Weigend 7697 (B)	W4024	Ackermann 610 (BSB)	KY286984	KY286715	KY286894	KY286804
Caiophora clavata	Ackermann 1102 (BONN)	W4516	Ackermann 1102 (BONN)	KY287002	KY286732	KY286912	KY286822
Caiophora coronata	Weigend 9152 (BSB)	W4009	Coccuci & Sersic 4845 (CORD)	KY286973	KY286704	KY286883	KY286793
Caiophora hibiscifolia	Ackermann 1103 (BONN)	W4030	Ackermann 1103 (BONN)	KY286988	KY286719	KY286898	KY286808
Caiophora lateritia	Ackermann 1104 (BONN)	W4031	Ackermann 1104 (BONN)	KY286989	KY286720	KY286899	KY286809
Caiophora stenocarpa	Ackermann et al. 758 (BSB)	W4015	Ackermann et al. 758 (BSB)	KY286978	KY286709	KY286888	KY286798
Grausa gayana	Weigend 7057 (MSB)	W1609	Weigend 7057 (MSB)	KY286962	KY286693	KY286872	KY286782
Huidobria fruticosa	Kern 6 (B)	W1327	Dillon 8034 (F)	KY286932	KY286664	KY286842	KY286752
Loasa acerifolia	Weigend 9142 (B)	W1340	Weigend et al. 6848 (BSB)	KY286937	KY286669	KY286847	KY286757
Loasa insons	Weigend 8724 (B)	W1360	Ackermann 536 (BSB)	KY286943	KY286674	KY286853	KY286763
Loasa nitida	Weigend et al. 7346 (BSB)	W1339	Weigend et al. 7346 (BSB)	KY286936	KY286668	KY286846	KY286756
Loasa sclareifolia	Weigend 8183 (B)	W4132	Weigend 8183 (B)	KY286994	KY286724	KY286904	KY286814
Loasa tricolor	Weigend 9010 (B)	W4016	Luebert 3021 (B)	KY286979	KY286710	KY286889	KY286799
Loasa triloba	Weigend 9008 (B)	W4511	Luebert & Bidart 3014b (B)	KY286999	KY286729	KY286909	KY286819
Nasa chenopodiifolia	Weigend 8433 (B)	W1415	Weigend et al. 7685 (M)	MF972162	MF972134	MF972176	MF972148
Nasa dillonii	Weigend 7556 (B)	W1413	Weigend 7556 (B)	MF972160	MF972132	MF972174	MF972146
Nasa dyeri subsp. australis	Dostert 98/80 (MSB)	W1624	Dostert 98/80 (MSB)	MF972165	MF972137	MF972179	MF972151
Nasa macrothyrsa	Weigend 7471 (BSB)	W1329	Weigend et al. 97/s.n. (M)	KY286934	KY286666	KY286844	KY286754
Nasa moroensis	Weigend 7694 (B)	W1414	Weigend 7694 (B)	MF972161	MF972133	MF972175	MF972147
Nasa moroensis	Weigend 8424 (B)	W2252	Weigend 8424 (B)	MF972167	MF972139	MF972181	MF972153
Nasa olmosiana	Dostert 98/163 (MSB)	W1567	Dostert 98/163 (MSB)	MF972163	MF972135	MF972177	MF972149
Nasa olmosiana	Weigend 8541 (BSB)	W4525	Weigend 8541 (BSB)	MF972171	MF972143	MF972185	MF972157
Nasa poissoniana subsp. glandulifera	Weigend & Schwarzer 8009 (B)	W4527	Weigend & Schwarzer 8009 (B)	MF972172	MF972144	MF972186	MF972158
Nasa poissoniana subsp. poissoniana	Ackermann 756 (BSB)	W1243	M. & K. Weigend 00/208 (NY)	KY286918	KY286650	KY286828	KY286738
Nasa ranunculifolia subsp. ranunculifolia	Henning 06/05 (B)	W2262	Henning 06/05 (B)	MF972169	MF972141	MF972183	MF972155
Nasa triphylla subsp. flavipes	Weigend & Dostert 98/203 (M)	W1623	Weigend & Dostert 98/203 (M)	MF972164	MF972136	MF972178	MF972150
Nasa triphylla subsp. triphylla	Ackermann 602 (BSB)	W2243	Ackermann 602 (BSB)	MF972166	MF972138	MF972180	MF972152
Nasa triphylla subsp. triphylla	Weigend & Brokamp 9098 (B)	W2680	Weigend & Brokamp 9098 (B)	MF972170	MF972142	MF972184	MF972156
Nasa vargasii	Weigend 5463 (B)	W2261	Weigend 5463 (B)	MF972168	MF972140	MF972182	MF972154
Nasa weigendii	Weigend 7913 (USM)	W4528	Weigend 7913 (USM)	MF972173	MF972145	MF972187	MF972159
Plakothira parviflora	Weigend s.n. (BSB)	W1290	Weigend s.n. (BSB)	KY286926	KY286658	KY286836	KY286746
Presliophytum heucheraefolium	Weigend 7691 (BONN)	W1369	Weigend 7368 (BSB)	KY286946	KY286677	KY286856	KY286766
Presliophytum incanum	Weigend & Dostert 97/12 (M)	W1288	Weigend & Förther 97/848 (F)	KY286924	KY286656	KY286834	KY286744
Scyphanthus elegans	Weigend 9032 (BSB)	W1467	Grau & Erhart 2-093 (M)	KY286958	KY286689	KY286868	KY286778
Scyphanthus stenocarpus	Gardner & Knees 8351 (BSB)	W4035	Gardner & Knees 8351 C (BSB)	KY286992	KY286723	KY286902	KY286812
Xylopodia klaprothioides	Weigend et al. 97/450 (M)	W1287	Weigend et al. 97/450 (M)	KY286923	KY286655	KY286833	KY286743
OUTGROUPS							
Deutzia discolor		W1293	Weigend 5615 (BSB)	KY286929	KY286661	KY286839	KY286749
Eucnide urens		W4499	Weigend 9153 (BSB)	KY286996	KY286726	KY286906	KY286816
Gronovia scandens		W4500	Weigend et al. 8522 (BSB)	KY286997	KY286727	KY286907	KY286817
Mentzelia albescens		W1285	Weigend et al. 6865 (BSB)	KY286921	KY286653	KY286831	KY286741
