# Supplementary material for: A case of behavioural diversification in male floral function – the evolution of thigmonastic pollen presentation
Source: Sci Rep. 2018 Sep 19;8:14018. doi: 10.1038/s41598-018-32384-4 (PMC6145904; doi:10.1038/s41598-018-32384-4)
Supplement: Supplementary file 3 — Supplementary Table 2 [file 41598_2018_32384_MOESM3_ESM.pdf]

A case of behavioral diversification in male floral function – the evolution of thigmonastic pollen presentation

Supplementary Table 2
Pollinator data

Tilo Henning, Moritz Mittelbach, Sascha A. Ismail, Rafael H. Acuña-Castillo, Maximilian Weigend

Taxon	Voucher plant	Literature	Pollination syndrome	Visitor taxon	Visitor family	Voucher visitor
Aosa rupestris Gardn.		1, 10		Gen. Spec.	Colletidae	
		15, 25		*Bicolletes nordestina* Urban, 2006	Paracolletini – Colletinae – Colletidae	
Blumenbachia insignis Schrad.		1, 5		Gen. Spec.	Colletidae	
		6		Gen. Spec.	Halictidae	
		1		Gen. Spec.	Megachilidae	
		15, 22		*Bicolletes pampeana*	Paracolletini – Colletinae – Colletidae	
		15, 22		*Colletes furfurascens*	Colletini - Colletinae– Colletidae	
		15, 22		*Colletes cyaneus*	Colletini – Colletinae – Colletidae	
		15, 22		*Dialictus opacus*	Halictini – Halictinae -Halictidae	
		15, 22		*Mielkeanthidium rubripes*	Anthidini – Anthidinae – Megachilidae?	
Blumenbachia latifolia St.Hil.		15, 16		Gen. Spec.	Colletidae	
		15		*Leioproctus fulvoniger*	Colletidae - ? - ?	
Caiophora arechavaletae (Urb.) Urb. & Gilg		15		*Bicolletes pampeana*	Paracolletini – Colletinae – Colletidae	
		15		*Bicolletes franki*	Paracolletini - Colletinae– Colletidae	
		15		*Chilicola spec.*	Paracolletini – Colletinae - Colletidae	
Caiophora canarinoides (Lenné & K.Koch) Urb. & Gilg		1		Hummingbird		
Caiophora carduifolia C.Presl.		1		Hummingbird		
Caiophora cernua (Griseb) Urb. & Gilg ex Kurtz		26		Gen. Spec.	Colletidae	
		4		Gen. Spec.	Apidae	
Caiophora chuquitensis (Meyen) Urb. & Gilg		26		Gen. Spec.	"Bee"	
		26		Hummingbird		
		4, 7, 26		Hummingbird		
		7		Gen. Spec.	Apidae	
		7, 15, 26		*Bombus spec.*	Apidae	
		7, 15		*Chilicola spec.*	Colletidae	
		7, 15		Gen. Spec.	Colletidae	
	M. & K. Weigend 2000/203			Hummingbird	Aves	Hummingbird observed
Caiophora cirsiifolia K.Presl	Henning & Schulz 22			*Lonchoryncha spec.*	Paracolletini - Colletidae	Henning & Schulz 33
				Centris spec.	Centridinii	Henning & Schulz 29
				Centris spec.	Centridinii	Henning & Schulz 31

				Gen. Spec.	?	Henning & Schulz 32
				Gen. Spec.	Megachilidae	Henning & Schulz 30
	Weigend et al. 7754			*Centris spec.*	Centridini - Apinae – Apidae	
				Centris spec.	Centridini - Apinae – Apidae	
		1		Bombus	Apidae	
		1, 20, 23		*Centris spec.*	Apidae	
	Weigend et al. 2003			*Centris spec.*	Centridini - Apinae – Apidae	
Caiophora clavata Urb. & Gilg		4		Fam. Gen. Spec.	"Bee"	
		7		*Bombus spec.*	Apidae	
		7, 15		*Chilicola*	Colletidae	
		7, 15, 26		Gen. Spec.	Colletidae	
		15		*Chilicola spec.*	Paracolletini - Colletidae – Colletina	
		15		*Colletes mastochila*	Colletini - Colletinae– Colletidae	
Caiophora coronata (Arn.) Hook & Arn.		1, 4, 26		Small rodent	Mammalia	
		1, 2, 4, 7		Hummingbird		
		1, 4		Passerines		
		1		Gen. Spec.	"Bee"	
		2, 12		*Bombus spec.*	Apidae	
		2, 4		Gen. Spec.	Megachilidae	
Caiophora hibiscifolia (Griseb.) Urb. & Gilg		26		Hummingbird		
		7		*Bombus spec.*	Apidae	
		7		Eucerini	Apidae	
		8		Hummingbird	Aves	
Caiophora lateritia (Hook.) Benth.		1, 4, 26		Hummingbird		
		14		Gen. Spec.	"Bee"	
		26		Gen. Spec.	Apidae	
		26		*Bombus spec.*	Apidae	
		26		*Xylocopa spec.*	Apidae	
		26		Gen. Spec.	Halictidae	
Loasa acerifolia Domb.		26		Gen. Spec.	"Bee"	
Loasa acerifolia Domb.	Weigend et al. 5848			*Bombus* cf. *dahlbomii*	Bombini – Apinae? – Apidae	
	Weigend et al. 6055			*Apis mellifera*	Apini – Apinae - Apidae	
				Bombus cf. *dahlbomii*	Bombini – Apinae? – Apidae	
Loasa insons Poepp.	Weigend et al. 5913			*Apis mellifera*	Apini – Apinae - Apidae	

				Gen. Spec.	Colletidae (1)	
				Gen. Spec.	Colletidae (2)	
				Gen. Spec.	Colletidae (3)	
	Weigend et al. 5913b			*Xylocopa spec.*	Xylocopini – Xylocopinae - Apidae	
				Apis mellifera	Apini – Apinae - Apidae	
				Gen. Spec.	Colletidae (2)	
				Gen. Spec.	Colletidae (1)	
				Tygathe spec.	Apidae (ehem.- Anthophoridae)	
Loasa nitida Desr.	Weigend 2000/660			Gen. Spec.	Megachilidae	
	Weigend et al. 2000/660			Gen. Spec.	Halictidae	
Loasa tricolor Ker Gawl.		3		Gen. Spec.	Apidae	
		3, 11, 12, 18, 20		Eucerini	Apidae	
		19		*Chilicola spec.*	Colletidae	
		3, 9, 10, 11, 12, 13, 18, 19, 24		Gen. Spec.	Colletidae	
		3, 11, 12		Gen. Spec.	Megachilidae	
		3, 11		Gen. Spec.	Halictidae	
		18			Diptera	
Nasa ranunculifolia (Kunth) Weigend	Weigend et al. 97/367			*Bombus* cf. *robustus*	Bombini – Apinae? – Apidae	97/Z1
Nasa ranunculifolia (*cymbopetala*)		21		Hummingbird		
Nasa dyeri (Urb. & Gilg) Weigend subsp. *australis* Dostert & Weigend	Dostert 98/80			*Apis mellifera*	Apinini – Apinae - Apidae	N. Dostert 1998/G.
				Trigona guianensis	? - Apidae	N. Dostert 1998/G.
				Partamona spec.	Meliponini - ? - Apidae	N. Dostert 1998/G.
Nasa macrothyrsa (Urb. & Gilg) Weigend	Henning & Schulz 32			*Neoxylocopa*	Xylocopini - Xylocopinae - Apidae	Henning & Schulz 39
				Apis mellifera	- Apidae	Henning & Schulz 40
				Neoxylocopa spec.	Xylocopini - Xylocopinae - Apidae	Henning & Schulz 41
				Apis mellifera	- Apidae	Henning & Schulz 42
	Weigend et al. 97/428			*Xylocopa viridigaster* Lepeletier 1843	Xylocopini - Xylocopinae - Apidae	
				Xylocopa lachnea (Neoxylocopa) Moure 1951	Xylocopini - Xylocopinae - Apidae	
	Henning & Schneider 118			Gen. Spec.	Colletidae	
				Xylocopa lachnea (Neoxylocopa) Moure 1951	Xylocopini - Xylocopinae - Apidae	

Nasa moroensis Weigend	Weigend et al. 7694			Gen. Spec.	Colletidae	
Nasa olmosiana (Gilg ex J.F. Macbr.) Weigend	Henning & Schulz 41			Gen. Spec.	?	Henning & Schulz 55
				Apis mellifera	Apidae	Henning & Schulz 56
				Apis mellifera	Apidae	Henning & Schulz 57
				Gen. Spec.	?	Henning & Schulz 58
				Gen. Spec.	?	Henning & Schulz 59
Nasa triphylla (Juss.) Weigend		1		Gen. Spec.	Colletidae	
		21		Gen. Spec.	Megachilidae	
Nasa urens (Jacq.) Weigend		1, 17		Gen. Spec.	Colletidae	
		21		Gen. Spec.	Megachilidae	
	Observation M. Weigend, no voucher			*Megachile spec.*	Megachilini – Megachilinae - Megachilidae	
				Gen. Spec.	Colletidae	
	Weigend & Skrabal 5889			Gen. Spec.	Colletidae (1)	
				Gen. Spec.	Colletidae (2)	
				Megachile spec.	Megachilini – Megachilinae - Megachilidae	
Nasa vargasii (Killip) Weigend	Weigend & Weigend 2000/396			Gen. Spec.	Megachilini – Megachilinae - Megachilidae	2000/396./Z1b. 2000/396./Z1c
				Caupolicana sp.	Caupolicanini – Diphaglossinae - Colletidae	2000/396./Z1b, 2000/396./Z1c
	Weigend & Weigend 2000/s.n.			Caupolicana sp.	Caupolicanini – Diphaglossinae - Colletidae	2000/s.n./Z1
Presliophytum heucheraefolium (Killip) Weigend		1		*Xylocopa spec.*	Apidae	
	Weigend et al. 5536			*Xylocopa spec.*	Xylocopini – Xylocopinae - Apidae	
				Megachile spec.	Megachilini – Megachilinae - Megachilidae	
Presliophytum incanum (Urb. & Gilg) Weigend	Observation M. Weigend, no voucher			*Xylocopa spec.*	Xylocopini – Xylocopinae - Apidae	
		1		Gen. Spec.	Colletidae	
		1			Lepidoptera	
	Weigend et al. 97/198			*Megachile spec.*	Megachilini – Megachilinae - Megachilidae	97/Z3
Scyphanthus elegans D.Don.		12		*Centris spec.*	Apidae	
		2		Eucerini	Apidae	
		2, 12		Gen. Spec.	Megachilidae	
Xylopodia klaprothioides	Henning & Schulz			Gen. Spec.	Colletidae - Colletini	Henning & Schulz 43

Weigend	34			Gen. Spec.	Colletidae - Colletini	Henning & Schulz 44
				Gen. Spec.	Colletidae - Colletini	Henning & Schulz 45
				Gen. Spec.	Gen. Spec. Diptera	Henning & Schulz 46
				Neoxylocopa spec.	Xylocopini - Xylocopinae - Apidae	Henning & Schulz 47
		1		Gen. Spec.	Colletidae	

Grey filling: data obtianed from the literature, wihout filling: own observations in the field. Color code for pollination syndrome:

short-tongued bees
long-tongued bees
hummingbirds
mammals
various (i.a. butterflies)

Literature:

1. Ackermann M, Weigend M (2006) Nectar, floral morphology and pollination syndrome in Loasaceae subfam. Loasoideae (Cornales). Ann. Bot. 98: 503-514
2. Arroyo MTK, Primack R, Armesto J (1982) Community studies in pollination ecology in the high temperate Andes of central Chile 1. Pollination mechanisms and altitudinal variation. Am. J. Bot. 69: 82-97.
3. Cares-Suarez R, Poch T, Acevedo RF, Acosta-Bravo I, Pimentel C, et al. (2011) Do pollinators respond in a dose-dependent manner to flower herbivory?: An experimental assessment in *Loasa tricolor* (Loasaceae). Gayana Bot. 68: 176-181.
4. Cocucci AA, Sérsic AN (1998) Evidence of rodent pollination in *Cajophora coronata* (Loasaceae). Pl. Syst. Evol. 211: 113-128.
5. Compagnucci LA, Roig Alsina A (2008) Nuevos *Leioproctus* Smith de la Argentina correspondientes a los subgéneros *Spinolapis* Moure y *Perditomorpha* Ashmead (Hymenoptera, Colletidae). Revista del Museo Argentino de Ciencias Naturales Nueva Serie 10: 319-327.
6. Devoto M (2006) Interacciones planta - polinizador a lo largo de un gradiente ambiental: una aproximación en escala de comunidad. Magister thesis. Buenos Aires, Argentina: Universidad de Buenos Aires. 130 p.
7. Harter B (1995) Blütenökologie einiger von Bienen und Kolibris bestäubter *Cajophora*-Arten (Loasaceae). Diploma thesis. Tübingen: University of Tübingen. 63 p.
8. Harter B, Schlindwein C, Wittmann D (1995) Bienen und Kolibris als Bestäuber von Blüten der Gattung *Cajophora* (Loasaceae). Apidologie 26: 356-357.
9. Jaffuel F, Pirion A (1926) Himenópteros del valle de Marga-Marga. Revista Chilena de Historia Natural 30: 362-383.
10. Martins CF (2002) Diversity of the Bee Fauna of the Brazilian Caatinga. In: Kevan P, Imperatriz Fonseca VL, editors. Pollinating Bees - The Conservation Link Between Agriculture and Nature. Brasilia: Ministry of Environment. pp. 131-134.
11. Montalva J, Castro B, Allendes JL (2010) Las Abejas del Jardín Botánico Chagual, estudio de caso de abejas nativas en zonas urbanas, Santiago Chile. Revista del Jardín Botánico Chagual 8: 13-23.
12. Montalva J, Vial P (2012) Abeja de Chile. (accessed 18.02.2014) http://abejasdechile.blogspot.de
13. Packer L, Zayed A, Grixti JC, Ruz L, Owen RE, et al. (2005) Conservation genetics of potentially endangered mutualisms: Reduced levels of genetic variation in specialist versus generalist bees. Conserv. Biol. 19: 195-202.
14. Sargent RD, Otto SP (2004) A phylogenetic analysis of pollination mode and the evolution of dichogamy in angiosperms. Evol. Ecol. Res. 6: 1183-1199.
15. Schlindwein C (2000) Verhaltensanpassungen oligolektischer Bienen an synchrone und an kontinuierliche Pollenpräsentation. In: Breckle SW, Schweizer B, Arndt U, editors. Ergebnisse weltweiter ökologischer Forschung. Stuttgart: Verlag Günter Heimbach. pp. 235-250.
16. Silveira FA (2009) A synopsis of Actenosigynes Moure, Graf & Urban, 1999 (Hymenoptera: Colletidae) - new species, possible oligolecty and biogeographic comments. Zootaxa: 15-24.
17. Stiles FG, Freeman CE (1993) Patterns in floral nectar characteristics of some bird-visited plant species from Costa Rica. Biotropica 25: 191-205.

18. Troncoso AJ, Vargas RR (2004) Efecto del vecindario floral sobre la tasa de visitas por insectos a *Loasa triloba* Domb. ex A.J. Juss. y *Loasa tricolor* Ker-Gawl En la Reserva Nacional de Río Clarillo, Región Metropolitana, Chile. Chloris chilensis 7: 1-8.
19. Urban D, Moure JS (2001) Catálogo de Apoidea da região. Neotropical (Hymenoptera, Colletidae). II. Diphaglossinae. Rev. Bras. Zool. 18: 1-34.
20. Vivallo F, Zanella FCV, Toro H (2003) Las especies chilenas de *Centris* (Paracentris) Cameron, 1903 y *Centris* (Penthemisia) Moure, 1950 (Hymenoptera: Apidae). In: Melo GAR, Alves-dos-Santos I, editors. Apoidea neotropica: Homenagem aos 90 anos de Jesus Santiago Moure. Santa Satarina: Editora Universidad do Extremo Sul Catarinense, Criciuma. pp. 77-83.
21. Weigend M, Gottschling M (2006) Evolution of funnel-revolver flowers and ornithophily in *Nasa* (Loasaceae). Pl. Biol. 8: 120-142.
22. Wittmann D, Schlindwein C (1995) Melittophilous plants, their pollen and flower visiting bees in Southern Brazil. 1. Loasaceae. Biociências 3: 19-34.
23. Zanella FCV (2002) Sistemática, filogenia e distribuição geográfica das espécies sul-americanas de *Centris* (Paracentris) Cameron, 1903 e de *Centris* (Penthemisia) Moure, 1950, incluindo uma análise filogenética do "grupo *Centris*" sensu Ayala, 1998 (Hymenoptera, Apoidea, Centridini). Rev. Bras. Entomol 46: 435-488.
24. Zayed A, Packer L, Grixti JC, Ruz L, Owen RE, et al. (2005) Increased genetic differentiation in a specialist versus a generalist bee: implications for conservation. Conservation Genetics 6: 1017-1026.
25. Leite AV, Nadia T, Machado IC (2016) Pollination of *Aosa rupestris* (Hook.) Weigend (Loasaceae): are stamen movements induced by pollinators? Braz. J. Bot. 39: 559–567.
26. Strelin MM, Benitez-Vieyra S, Fornoni J, Klingenberg CP, Cocucci AA (2016) Exploring the ontogenetic scaling hypothesis during the diversification of pollination syndromes in *Caiophora* (Loasaceae, subfam. Loasoideae). Ann. Bot. 117: 937-947.
