# Supplementary material for: A case of behavioural diversification in male floral function – the evolution of thigmonastic pollen presentation
Source: Sci Rep. 2018 Sep 19;8:14018. doi: 10.1038/s41598-018-32384-4 (PMC6145904; doi:10.1038/s41598-018-32384-4)
Supplement: Supplementary file 4 — Supplementary Material 3 [file 41598_2018_32384_MOESM4_ESM.pdf]

A case of behavioral diversification in male floral function – the evolution of thigmonastic pollen presentation

Supplementary Material 3

Tilo Henning, Moritz Mittelbach, Sascha A. Ismail, Rafael H. Acuña-Castillo, Maximilian Weigend

Contents

- Additional figure 2
- Data Exploration 3
- Generalized Additive Models (GAMs) 6
- Generalized Additive Mixed Models (GAMMs) 9
  - Defining the (best) model 9
  - Selection of terms 10
  - Model Summary 11
  - Modell validation 15
- Case study: short-tongued bees 20
  - GAMM 20
  - Model validation 22
- Phylogenetic Signal 23
- References 25

Additional figure

Figure 1 shows average thigmonastic patterns of sampled plants assigned to pollination syndromes (solid lines) and respective controls (dotted lines).

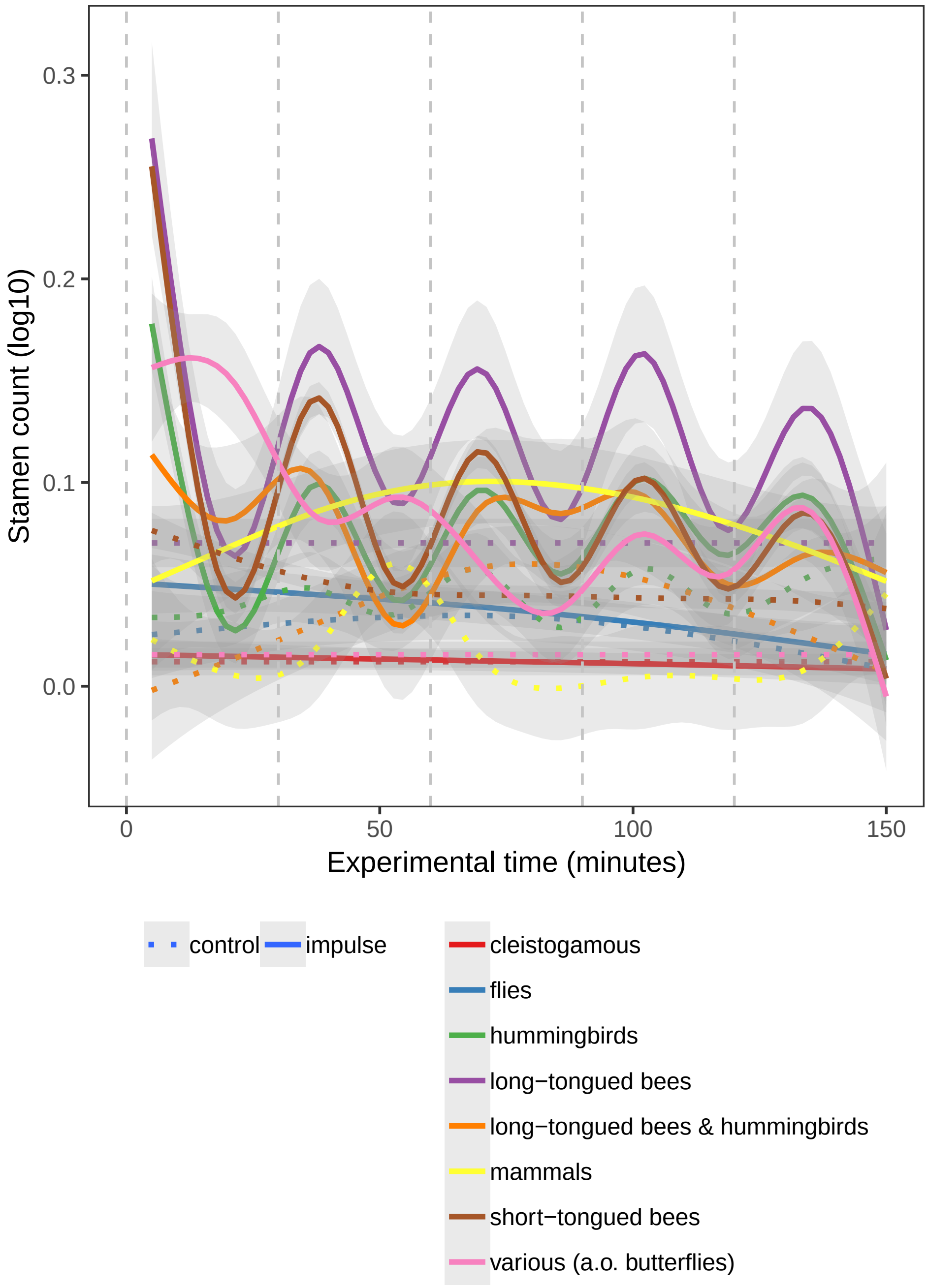

Figure 1: Differences in staminal movements between genera during experimental time in reaction to manual stimulations of the floral organs. Dashed horizontal lines mark stimulations. First evaluation of staminal reaction occured 5 minutes after stimulus. Solid lines are estimated smooths colored for each genus. Shaded ribbons show 95% confidence intervals.

Data Exploration

The data exploration follows guidelines by Zuur, Alain et al. (2009) and Zuur (2012).
1. We do not find serious **colinearity** between covariates (Figure 2)(except for *time.absolute* and *impulse.period*, but those two won't be used together anyway).

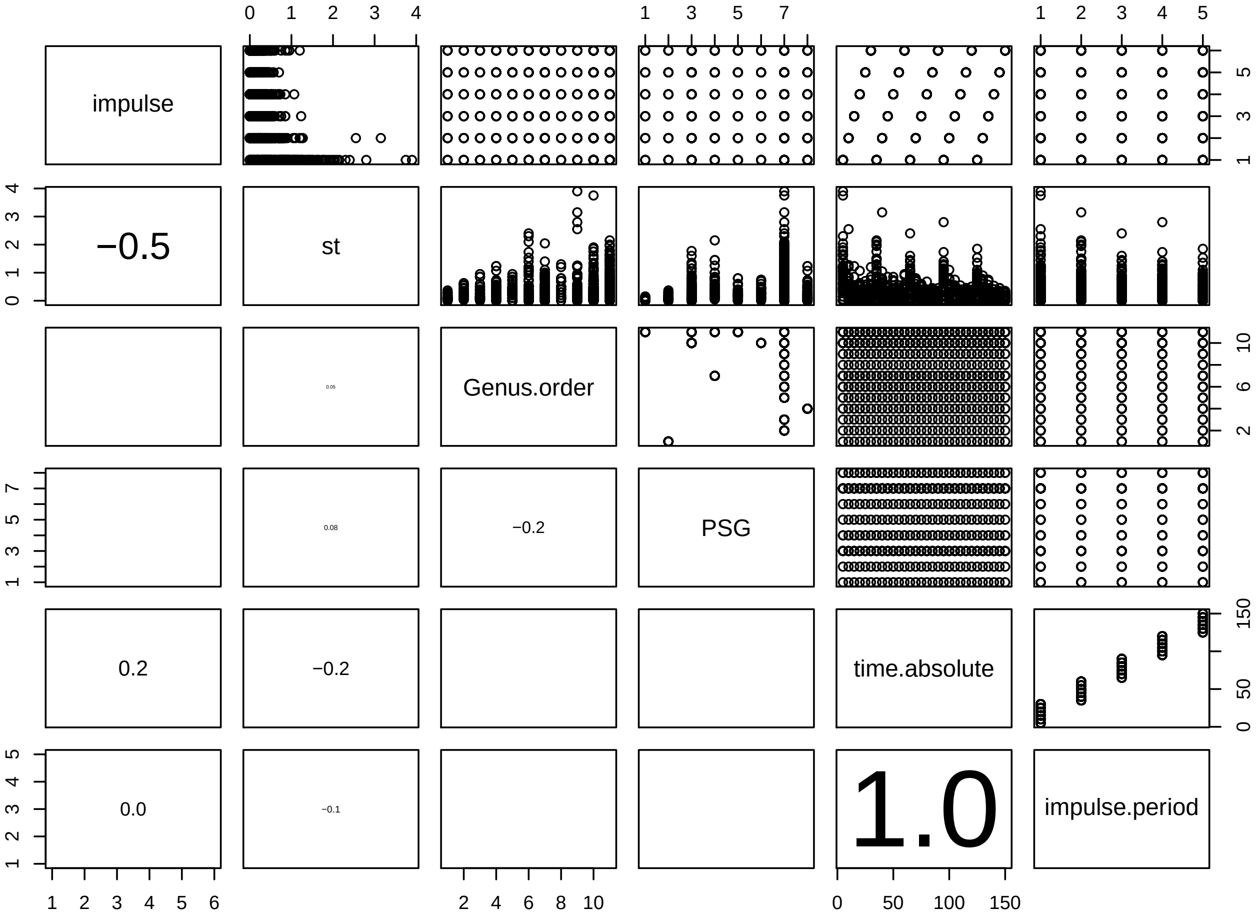

Figure 2: Pairwise correlations of covariates.

2. We test the assumption of **heterogeneity** within categories of the data (Figure 3-6). We check whether the response variable is balanced with respect to the categorical covariates, and find marginal differences among factor levels. All variables should be included in the fixed part of the model (again: only one of time.absolute and impulse.period should be used).

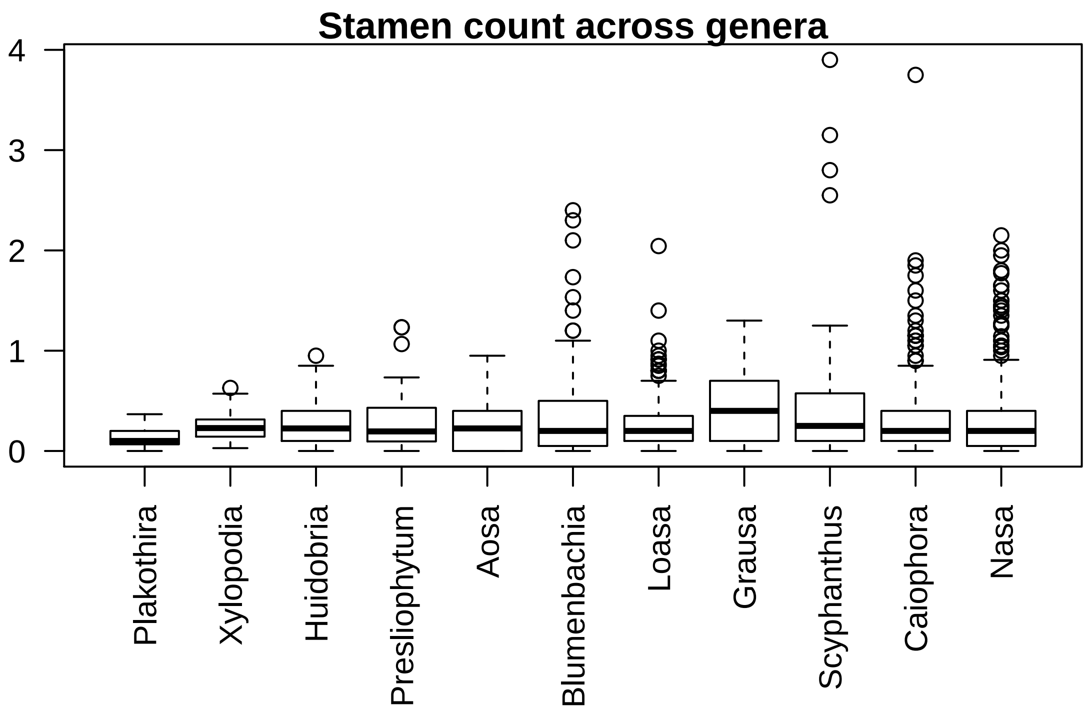

Figure 3: Stamen count across genera.

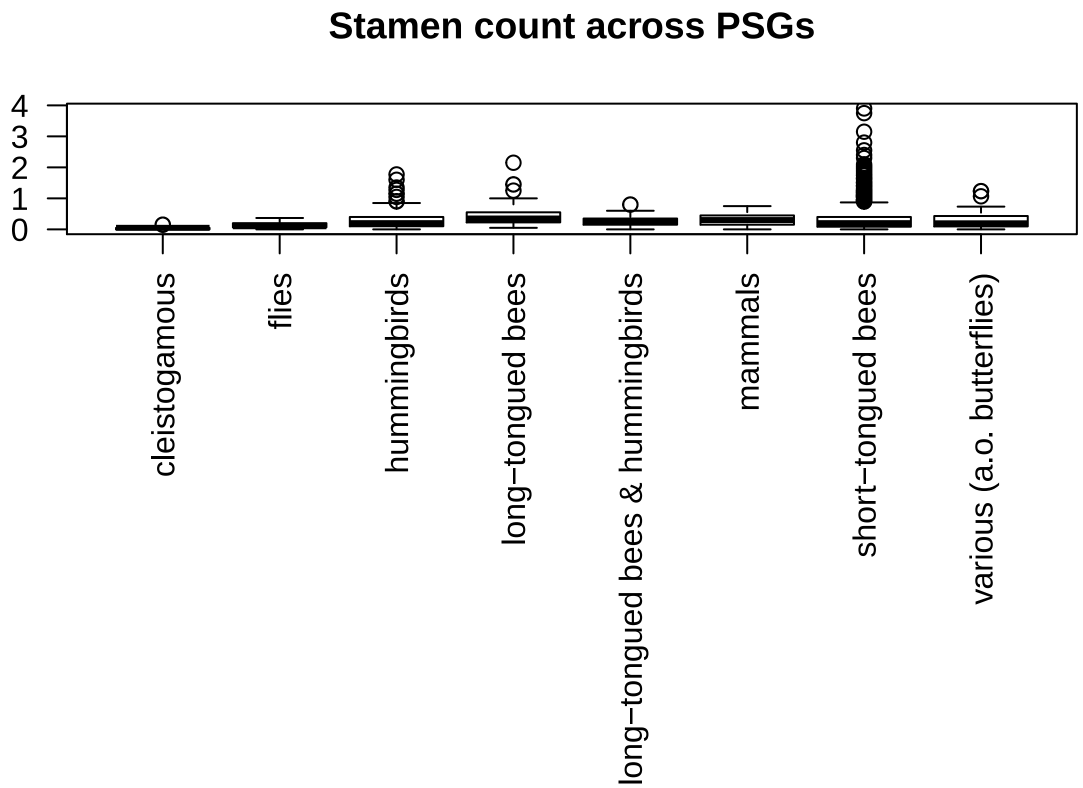

Figure 4: Stamen count across pollination syndrome groups (PSGs)

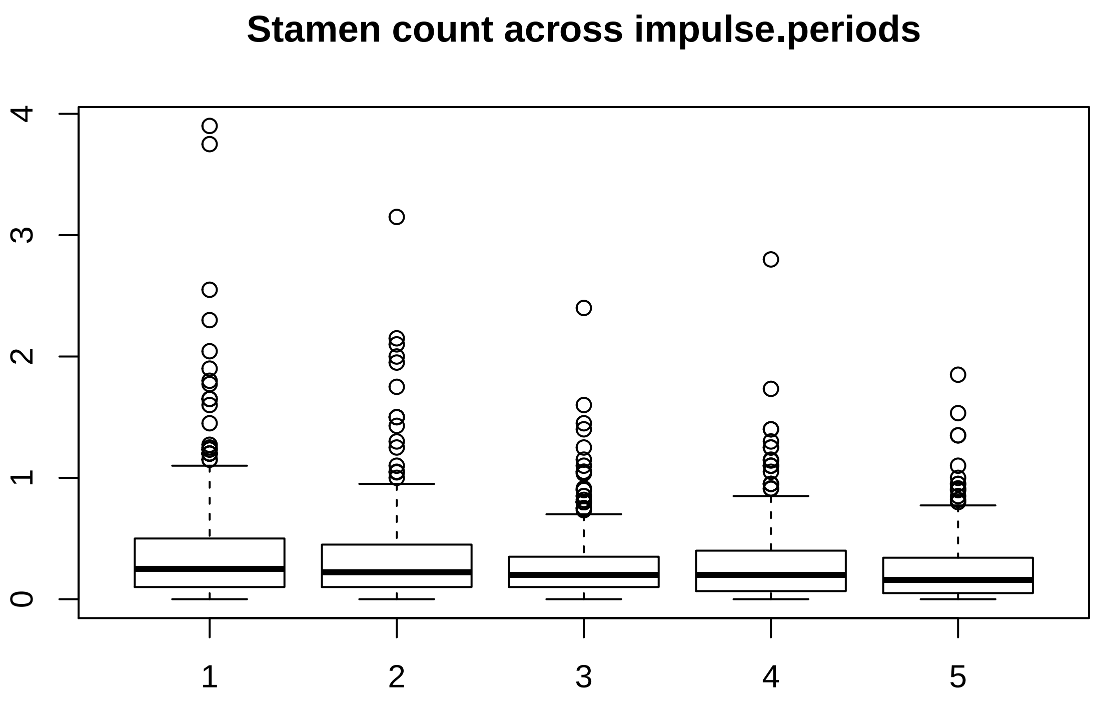

Figure 5: Stamen count across impuls periods

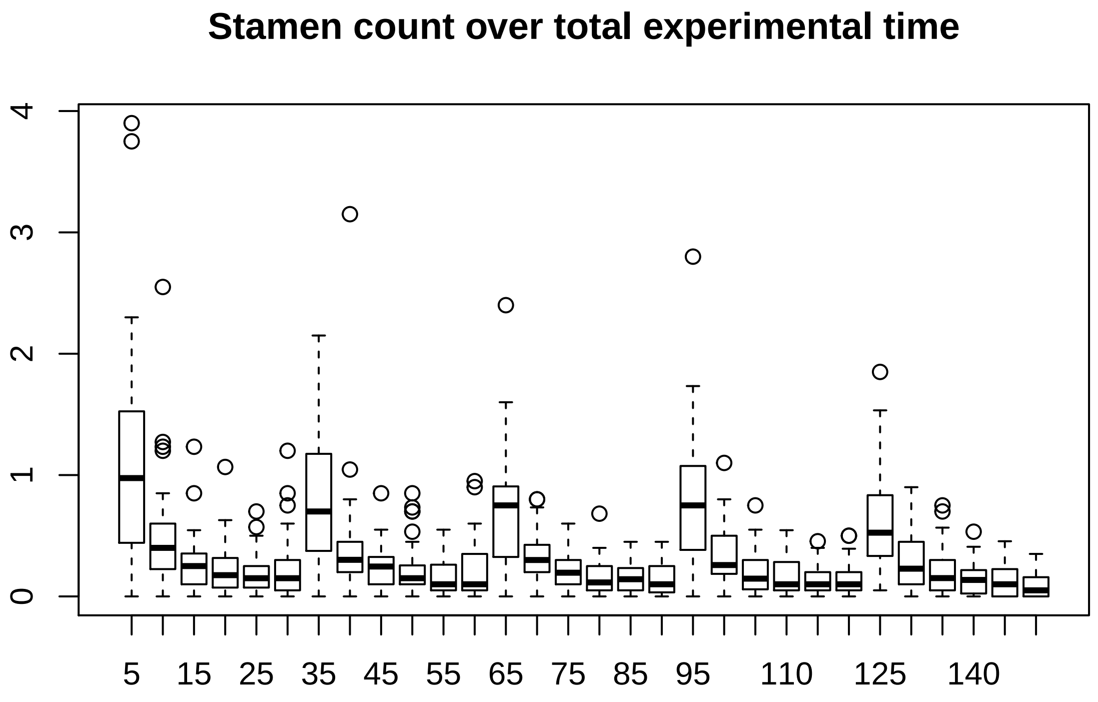

Figure 6: Stamen count over experimental time.

3. The dotplot shows two **outliers**, but we decide to use all observations in the analysis (Figure 7).

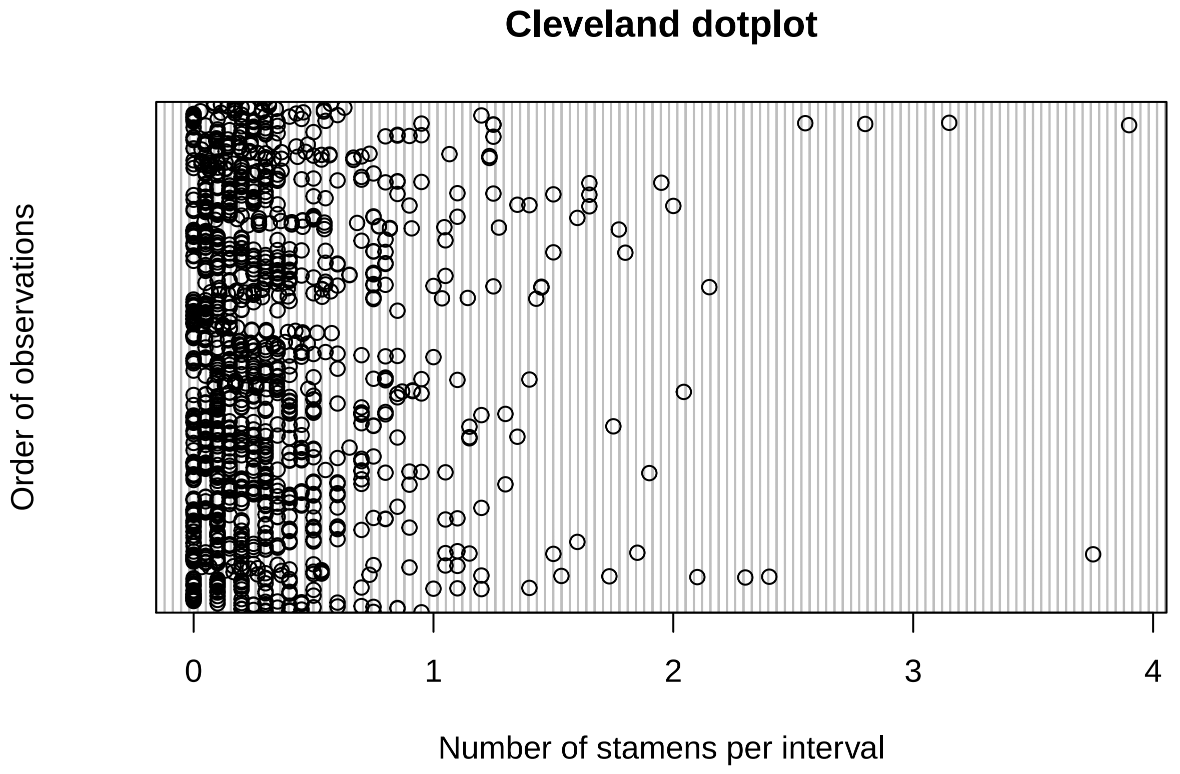

Figure 7: Cleveland dotplot.

Generalized Additive Models (GAMs)

Do stamens move in reaction to manual impulses?

To answer this question, we applied pairwise GAMs as implemented in the mgcv package for each sampled species individually (Wood 2011). We used the average number of moving stamens over all *impulse.periods* as response variable, predicted by the impulse. Thereby, we allowed for different smooths for each levels of treatment ("control", "Impulse"). Subsequently, we controlled for a *false discovery rate* (FDR) using the Benjamini-Hochberg procedure (Benjamini and Hochberg 1995).Table 1 shows the adjusted *p-values* (*p.adjusted*) for the manipulated flowers of each sampled species in comparison to the respective controls. Since we controlled for the FDR, we applied a significance cut-off at *p<=0.05* (in bold). Resulting smooth terms of single GAMs are plotted in Figure 8. Panels with green headers present significant thigmonasty.

Table 1: Species wise comparisons of stamen movement between experimentally stimulated and control flowers.

label	edf	Fv	p.adj
A . parviflora	4.443	6.058	**0.001**
A . rupestris	1.000	7.263	0.051
B . hieronymie	3.300	23.515	**0.000**
B . insignis	3.062	59.432	**0.000**
B . latifolia	2.330	37.104	**0.000**
C . arechavalatae	3.199	60.296	**0.000**
C . canarinoides	1.001	18.536	**0.000**
C . carduifolia	2.294	9.063	**0.000**
C . cernua	1.269	51.023	**0.000**
C. cf. andina	2.138	18.723	**0.000**
C . chuquitensis	1.000	14.192	**0.002**
C . cirsifolia	1.436	15.849	**0.000**
C . clavata	1.000	125.883	**0.000**
C . coronata	1.000	5.445	0.1
C . hibiscifolia	1.000	18.846	**0.000**
C . lateritia	2.241	43.235	**0.000**
C . stenocarpa	2.169	40.254	**0.000**
G . gayana	1.000	31.097	**0.000**
H . fruticosa	1.000	5.110	0.1
L . acerifolia	3.124	36.000	**0.000**
L . insons	1.863	30.387	**0.000**
L . nitida	1.000	21.381	**0.000**
L . sclareifolia	1.000	28.320	**0.000**
L . tricolor	2.648	12.012	**0.000**
L . triloba	3.696	20.121	**0.000**
N . chenopodiifolia	1.001	0.675	0.481
N . dillonii	2.441	9.369	**0.000**
N. dyeri subsp. australis	2.786	35.142	**0.000**
N . macrothyrsa	2.407	35.097	**0.000**
N . moroensis	1.217	21.115	**0.000**
N . olmosiana	3.256	12.271	**0.000**
N. poissoniana subsp. glandulifera	2.386	66.373	**0.000**
N. poissoniana subsp. poissoniana	1.840	40.327	**0.000**
N. ranunculifolia subsp. ranunculifolia	1.000	32.153	**0.000**
N. triphylla subsp. flavipes	2.248	39.679	**0.000**
N. triphylla subsp. triphylla	3.415	56.229	**0.000**
N . vargasii	2.907	50.251	**0.000**
N . weigendii	2.081	38.804	**0.000**
P . parviflora	4.174	5.870	**0.000**
P . heucheraefolium	2.443	13.494	**0.000**
P . incanum	1.818	2.945	0.177
S . elegans	2.045	18.359	**0.000**
S . stenocarpus	3.780	41.832	**0.000**
X . klaprothioides	1.000	0.498	0.481

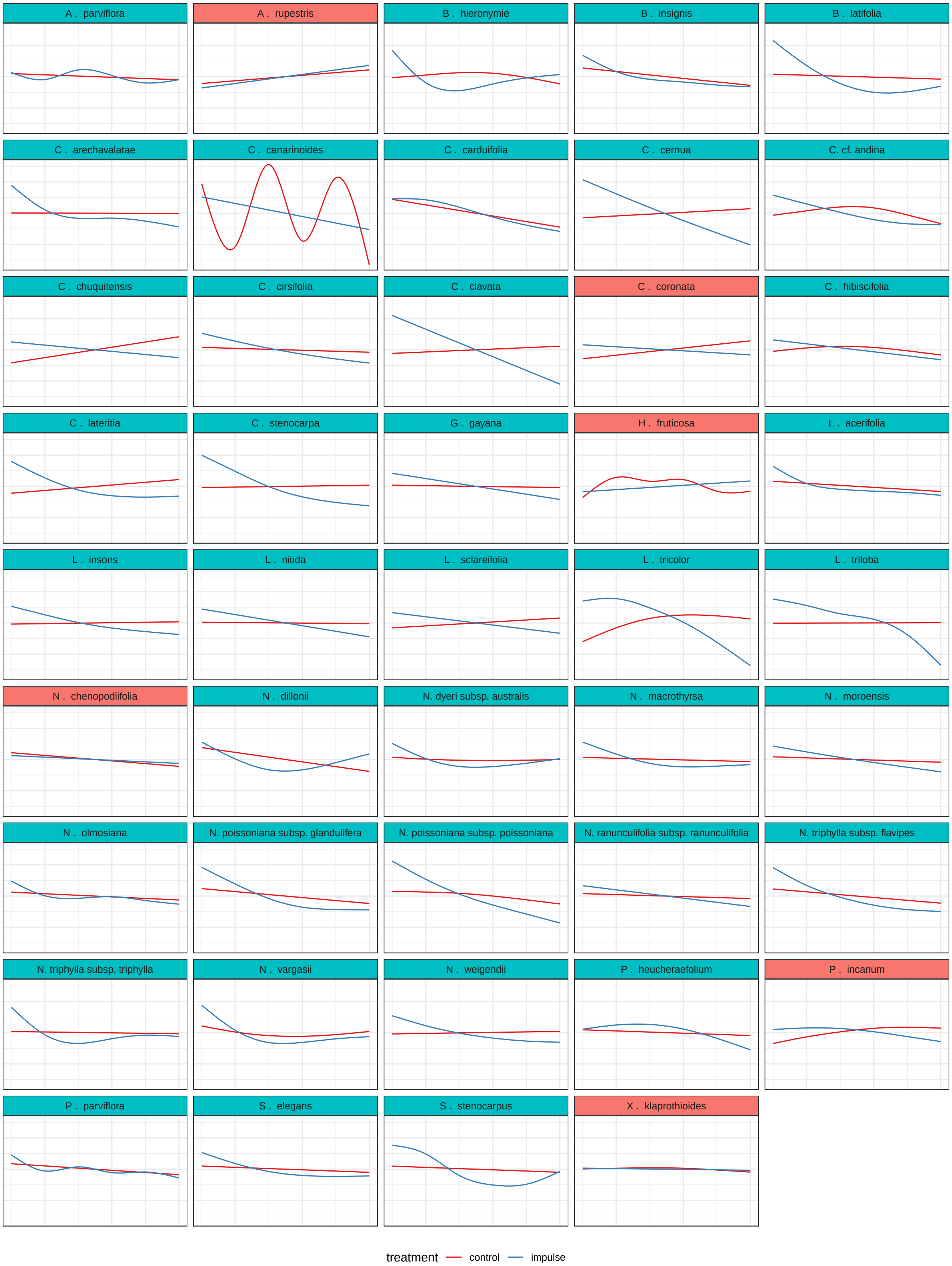

Figure 8: Smoothers of single GAMs. Red headers mark non-significant, green headers significant differences between treatment and control at *p<=0.05*. Controls are printed in red lines, treatment in blue lines.

Generalized Additive Mixed Models (GAMMs)

2. Do plants across different clades within the Loasaceae family respond to predominant pollinator-groups through the adaptation of stamen movement profiles? And if so, are these patterns pollinator specific?

Defining the (best) model

Fixed term

We focus this analysis on the time frame of stamen counts after one single impulse (30min). By adding the factor variable PSG (pollination syndrome group) to the smoother, we allow a replicate smooth per factor level see p.53 in Zuur et al. 2014. Since these smooths will be centered, the factor needs to be added as a main effect as well. We define the modelling formula as follows:

mean.stamens ~ s(impulse,k=6,by=PSG) + PSG + s(dist) + s(impulse.period,k=5)

Random factors

To account for different reaction shapes in different species, we add the species ID as random factor:

random=list(species=~1)

Distribution and Link

Since we have strictly positive counts, Poisson was our first choice. But the models were underdispered and we re-calculated the best model using a quasipoisson distribution with a log-link function, which work out well.

Temporal auto-correlation

We add a temporal auto-correlation to account for the time series (impulse A to E), which is nested in repeated measures (*impulse.period*: 1:5):

correlation=corCAR1(form=~1|impulse.period)

Phylogenetic correlation

Pylogenetic correlation is calculated based on tree architecture using the "nNodes" argument in the distRoot-function of the "adephylo" package (Jombart, Balloux, and Dray 2010)

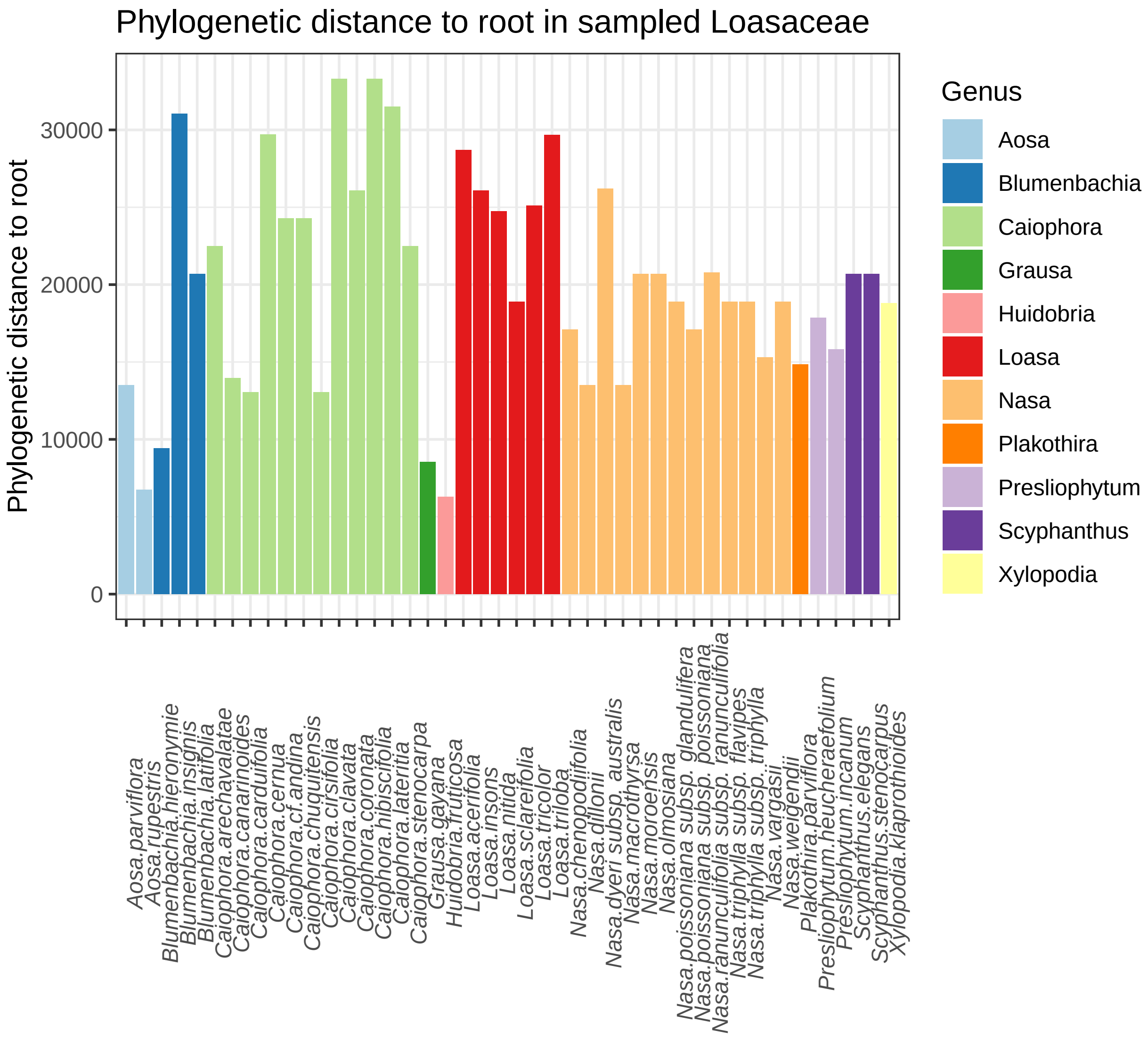

Figure 9: Phylogenetic distance of each species to root of the phylogenetic tree. Colors represent different genera.

Selection of terms

To select the **best model**, we apply the dredge function as implemented in the package: MuMIn.We choose the full model (as initially proposed on basis of scientific expectation and formulated hypothesis), although dredging reveals that the parametric factor *PSG* and the smooth *s(dist)* should be excluded. Nonetheless, the ranks are ordered according to *AICc* and the *logliklihood* on the other hand shows a slight preference for the full model.

Table 2: Results of automated model selection with dredge()

(Intercept)	PSG	s(dist)	s(impulse k=6 by = PSG)	s(impulse.period k=5)	df	logLik	AICc	delta	weight
-1.46	NA	NA	+	+	22	-1562.01	3168.79	0.00	0.94
-1.46	NA	+	+	+	24	-1563.16	3175.25	6.46	0.04
-3.21	+	NA	+	+	29	-1558.28	3175.92	7.12	0.03
-3.19	+	+	+	+	31	-1559.27	3182.07	13.28	0.00
-1.44	NA	NA	+	NA	20	-1586.91	3214.47	45.68	0.00
-3.19	+	NA	+	NA	27	-1585.14	3225.45	56.65	0.00
-3.16	+	+	+	NA	29	-1586.45	3232.24	63.45	0.00
-1.12	NA	NA	NA	+	6	-1791.51	3595.08	426.28	0.00
-1.12	NA	+	NA	+	8	-1791.19	3598.49	429.69	0.00
-1.09	NA	+	NA	NA	6	-1794.20	3600.47	431.67	0.00
-3.16	+	NA	NA	+	13	-1801.82	3629.91	461.12	0.00
-3.15	+	+	NA	+	15	-1801.53	3633.42	464.63	0.00
-3.14	+	NA	NA	NA	11	-1806.62	3635.44	466.64	0.00
-3.12	+	+	NA	NA	13	-1806.29	3638.87	470.07	0.00

Model Summary

```
Family: quasipoisson
Link function: log

Formula:
st ~ s(impulse, k = 6, by = PSG) + PSG + s(dist) + s(impulse.period,
    k = 5)

Parametric coefficients:
                                    Estimate Std. Error t value Pr(>|t|)
(Intercept)                          -3.1899     0.5881  -5.424 6.93e-08
PSGflies                              1.0446     0.7517   1.390 0.164881
PSGhummingbirds                       1.6861     0.6163   2.736 0.006306
PSGlong-tongued bees                  2.2818     0.6544   3.487 0.000505
PSGlong-tongued bees & hummingbirds   1.7956     0.7240   2.480 0.013253
PSGmammals                            2.0148     0.7289   2.764 0.005785
PSGshort-tongued bees                 1.7623     0.5931   2.972 0.003018
PSGvarious (a.o. butterflies)         1.7418     0.6607   2.636 0.008478

(Intercept)                         ***
PSGflies
PSGhummingbirds                     **
PSGlong-tongued bees                ***
PSGlong-tongued bees & hummingbirds *
PSGmammals                          **
PSGshort-tongued bees               **
PSGvarious (a.o. butterflies)       **
---
Signif. codes:  0 '***' 0.001 '**' 0.01 '*' 0.05 '.' 0.1 ' ' 1
```

```
Approximate significance of smooth terms:
                                               edf Ref.df       F
s(impulse):PSGcleistogamous                  1.000  1.000   0.265
s(impulse):PSGflies                          1.000  1.000   5.428
s(impulse):PSGhummingbirds                   2.480  2.480  36.844
s(impulse):PSGlong-tongued bees              2.405  2.405  20.413
s(impulse):PSGlong-tongued bees & hummingbirds 1.645  1.645   6.438
s(impulse):PSGmammals                        1.000  1.000   1.967
s(impulse):PSGshort-tongued bees             4.397  4.397 166.226
s(impulse):PSGvarious (a.o. butterflies)     2.602  2.602   9.692
s(dist)                                      1.000  1.000   0.197
s(impulse.period)                            1.000  1.000  45.015
                                              p-value
s(impulse):PSGcleistogamous                   0.60649
s(impulse):PSGflies                           0.01997 *
s(impulse):PSGhummingbirds                    < 2e-16 ***
s(impulse):PSGlong-tongued bees              2.22e-10 ***
s(impulse):PSGlong-tongued bees & hummingbirds  0.00211 **
s(impulse):PSGmammals                         0.16099
s(impulse):PSGshort-tongued bees              < 2e-16 ***
s(impulse):PSGvarious (a.o. butterflies)     2.53e-05 ***
s(dist)                                       0.65723
s(impulse.period)                            2.87e-11 ***
---
Signif. codes:  0 '***' 0.001 '**' 0.01 '*' 0.05 '.' 0.1 ' ' 1

R-sq.(adj) =  0.435
  Scale est. = 0.14272   n = 1320
```

The $gam part of the model reveals highly significant *smooth* (when compared to a straight line, ergo the idealized control) except for *cleistogamous* flowers and flowers visited by *flies* and *mammals*. The phylogenetic distance has no effect. The impact of the *impulse.period* might be an artifact of the repeated sampling design. The *parametric coefficients* show high impacts of *Long-tongued bees*. Nonetheless, the subsequent *ANOVA* shows that the global effect of *PSG* is not significant:

```
Family: quasipoisson
Link function: log

Formula:
st ~ s(impulse, k = 6, by = PSG) + PSG + s(dist) + s(impulse.period,
    k = 5)

Parametric Terms:
    df     F p-value
PSG  7 2.163  0.0349

Approximate significance of smooth terms:
                                               edf Ref.df       F
s(impulse):PSGcleistogamous                  1.000  1.000   0.265
s(impulse):PSGflies                          1.000  1.000   5.428
s(impulse):PSGhummingbirds                   2.480  2.480  36.844
s(impulse):PSGlong-tongued bees              2.405  2.405  20.413
s(impulse):PSGlong-tongued bees & hummingbirds 1.645  1.645   6.438
```

```
s(impulse):PSGmammals                         1.000  1.000   1.967
s(impulse):PSGshort-tongued bees              4.397  4.397 166.226
s(impulse):PSGvarious (a.o. butterflies)      2.602  2.602   9.692
s(dist)                                       1.000  1.000   0.197
s(impulse.period)                             1.000  1.000  45.015
                                               p-value
s(impulse):PSGcleistogamous                    0.60649
s(impulse):PSGflies                            0.01997
s(impulse):PSGhummingbirds                     < 2e-16
s(impulse):PSGlong-tongued bees               2.22e-10
s(impulse):PSGlong-tongued bees & hummingbirds  0.00211
s(impulse):PSGmammals                          0.16099
s(impulse):PSGshort-tongued bees               < 2e-16
s(impulse):PSGvarious (a.o. butterflies)      2.53e-05
s(dist)                                        0.65723
s(impulse.period)                             2.87e-11
```

The smooth shapes show the non-linearity (here: thigmonasty).

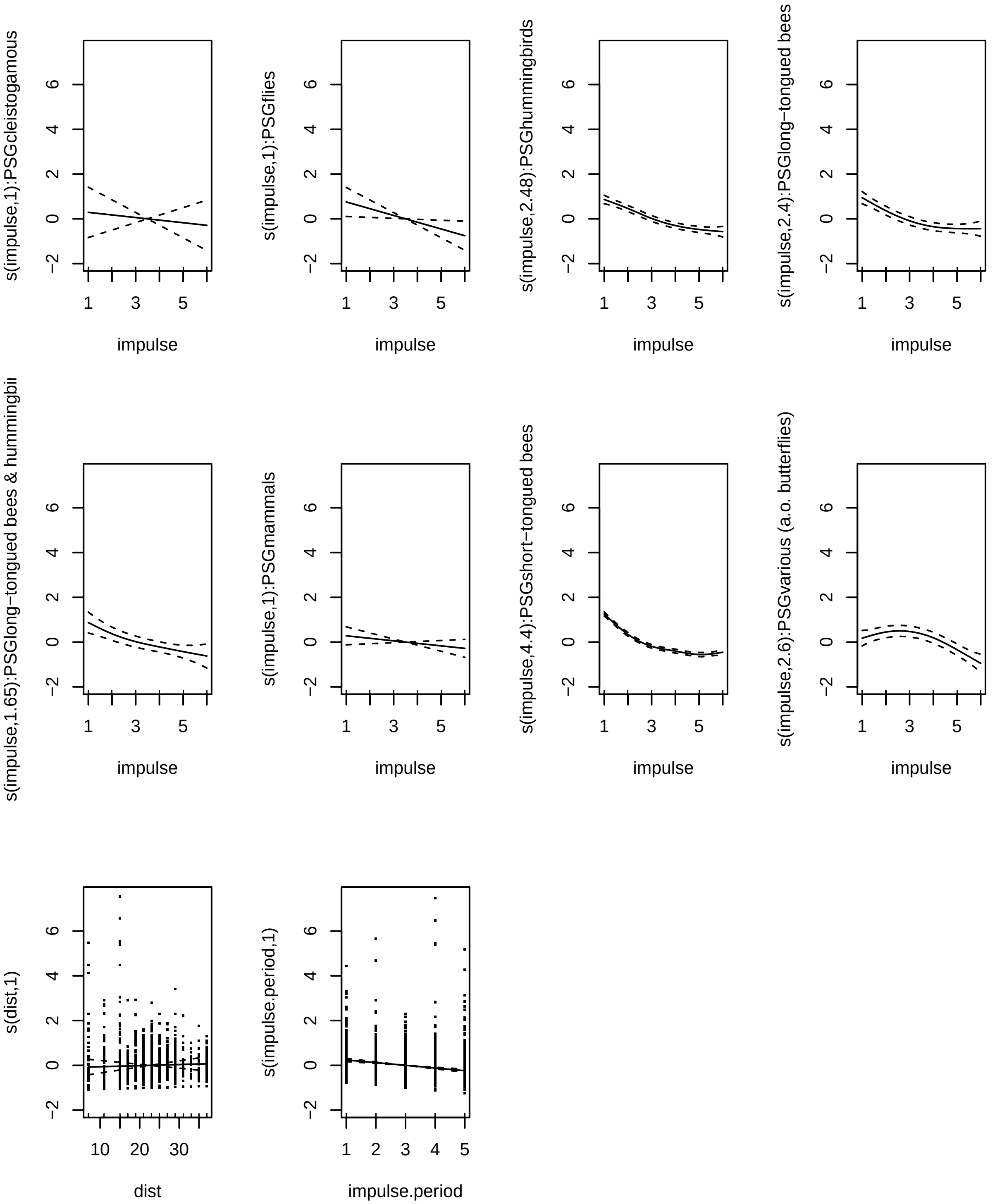

Figure 10: Smoother of each PSG as estimated in GAMM.

Modell validation

Overdispersion

The over-dispersion is calculated as: 0.96. The following plots of pearson-residuals should not show any patterns. (It is not perfect, but I think the best we can do here…):

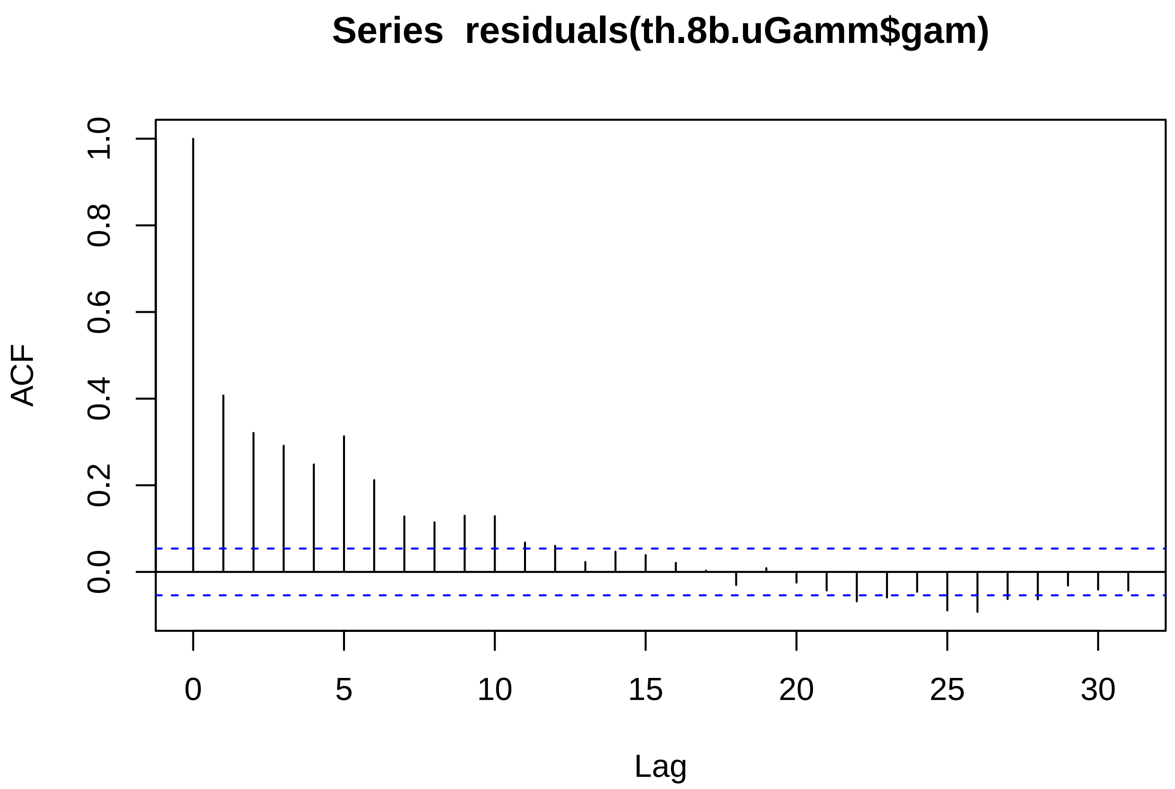

Figure 11: Plots of GAMM residuals against different predictors and factors.

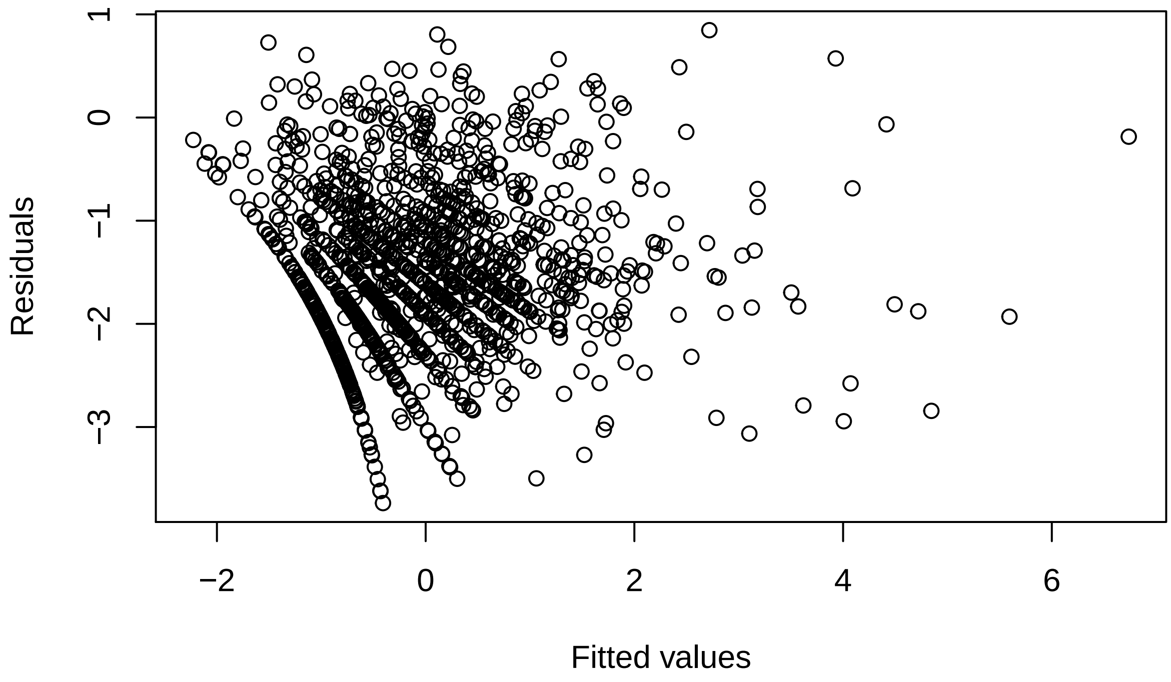

Figure 12: Plots of GAMM residuals against different predictors and factors.

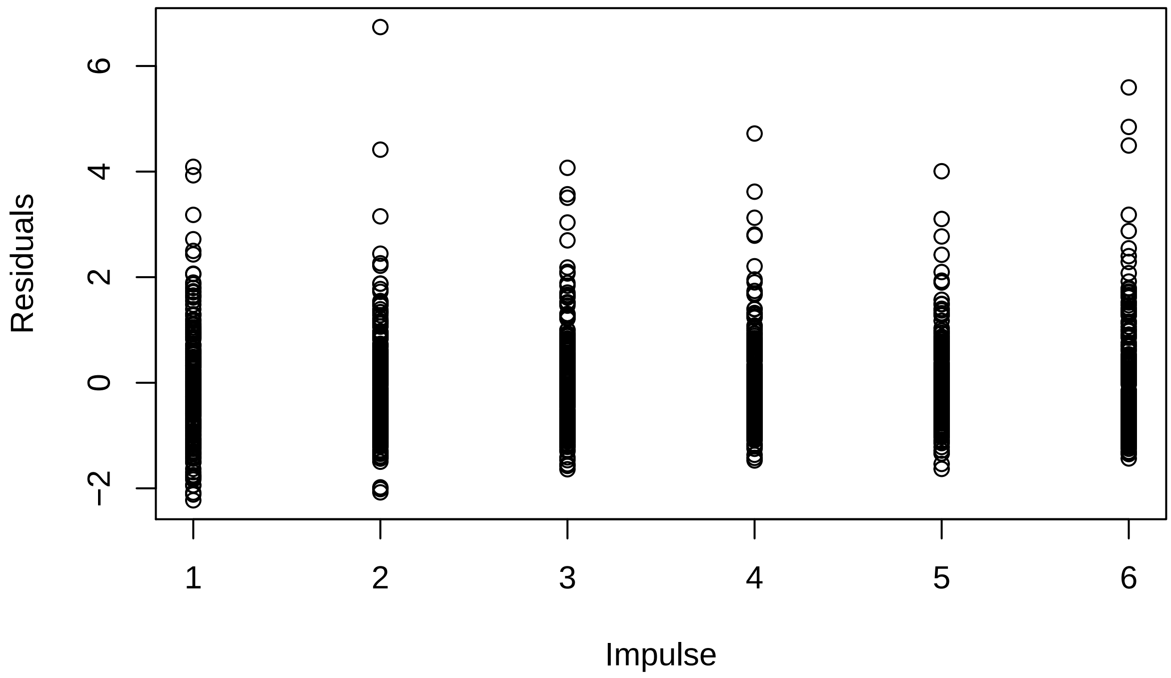

Figure 13: Plots of GAMM residuals against different predictors and factors.

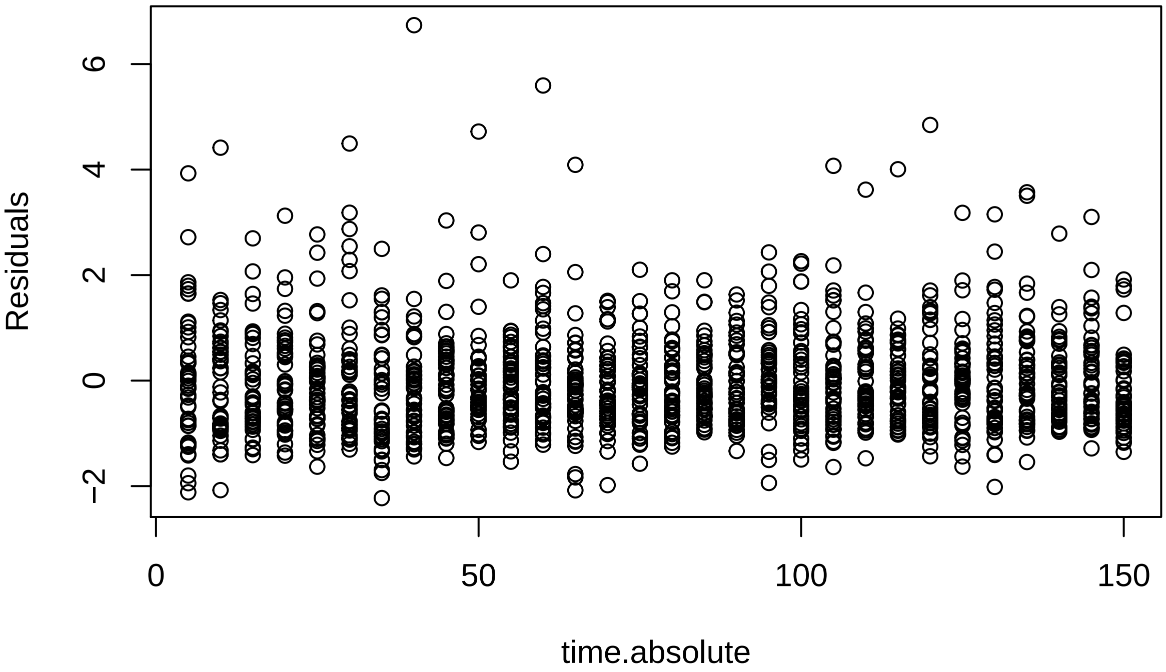

Figure 14: Plots of GAMM residuals against different predictors and factors.

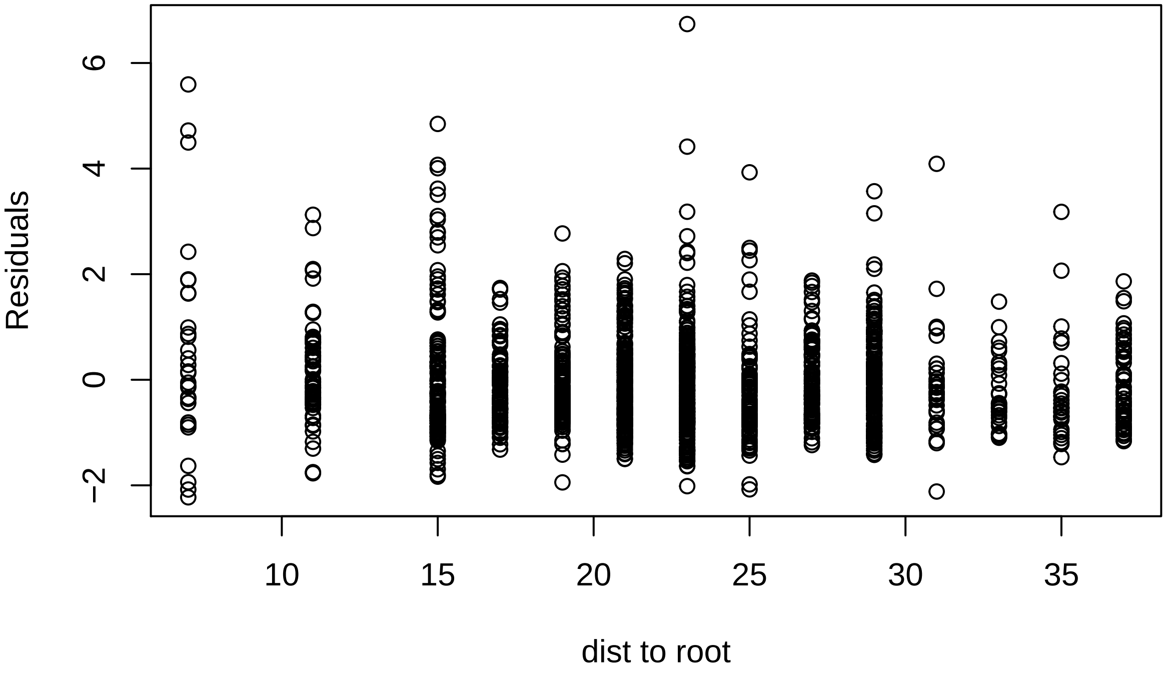

Figure 15: Plots of GAMM residuals against different predictors and factors.

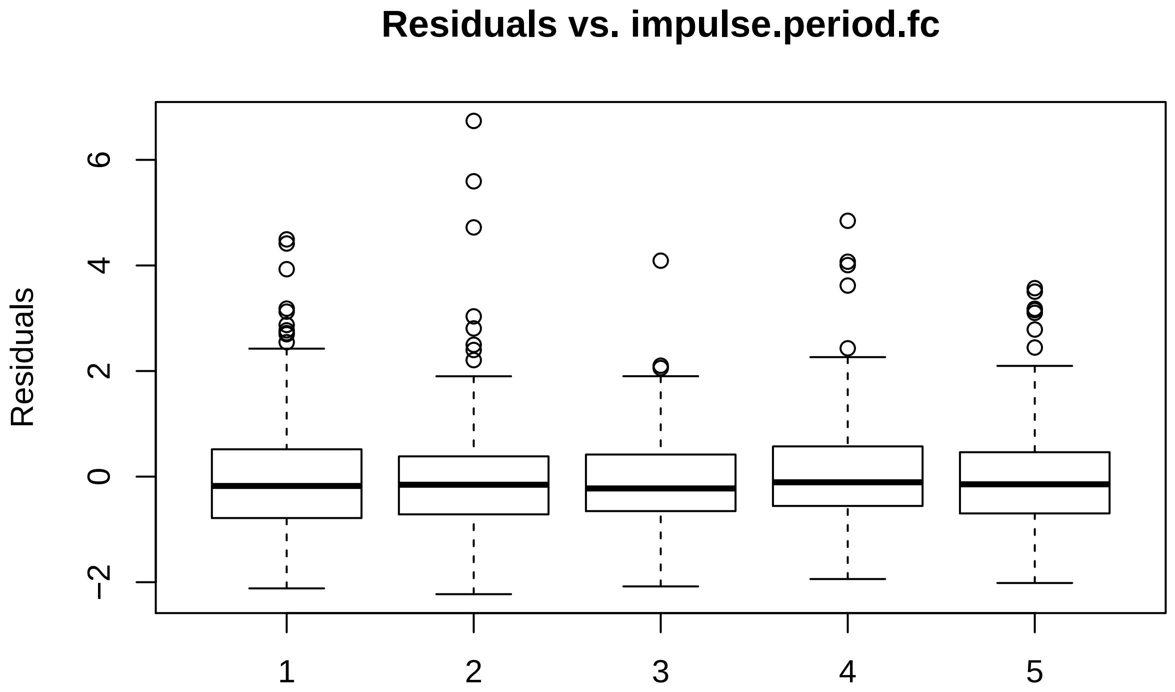

Figure 16: Plots of GAMM residuals against different predictors and factors.

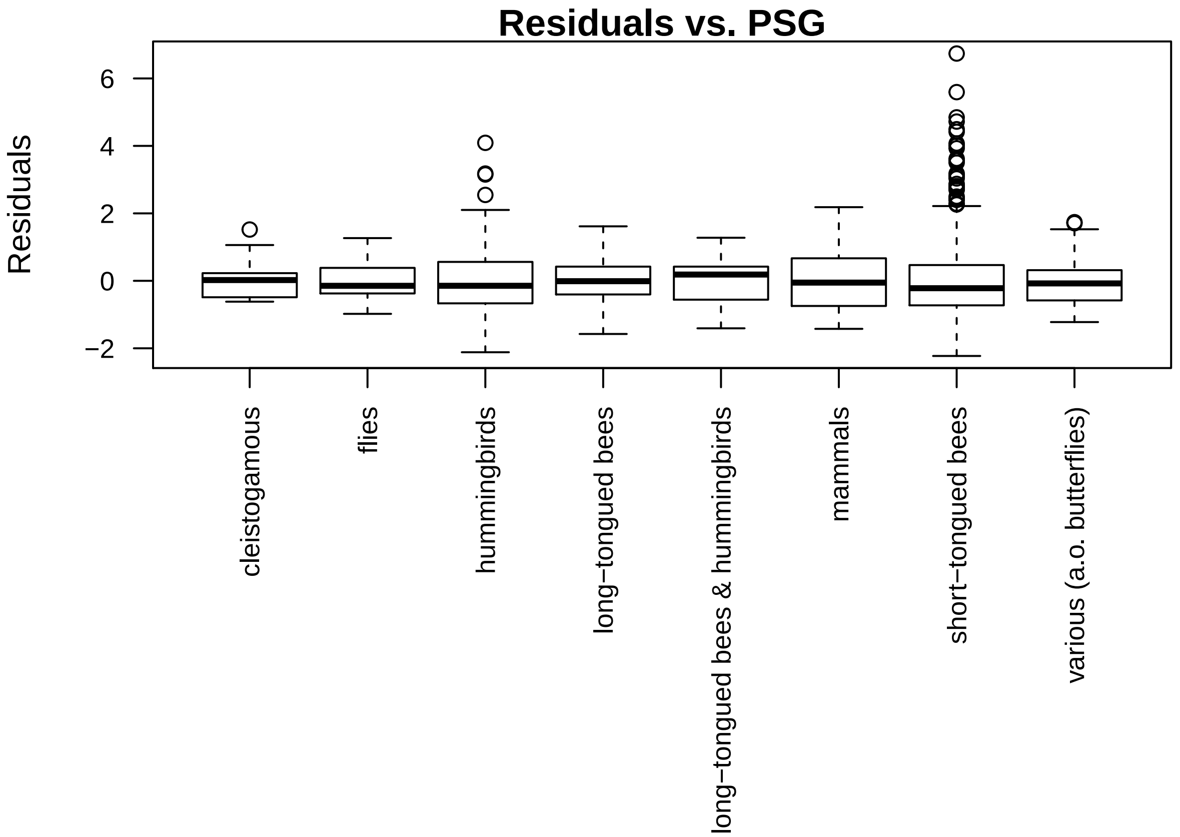

Figure 17: Plots of GAMM residuals against different predictors and factors.

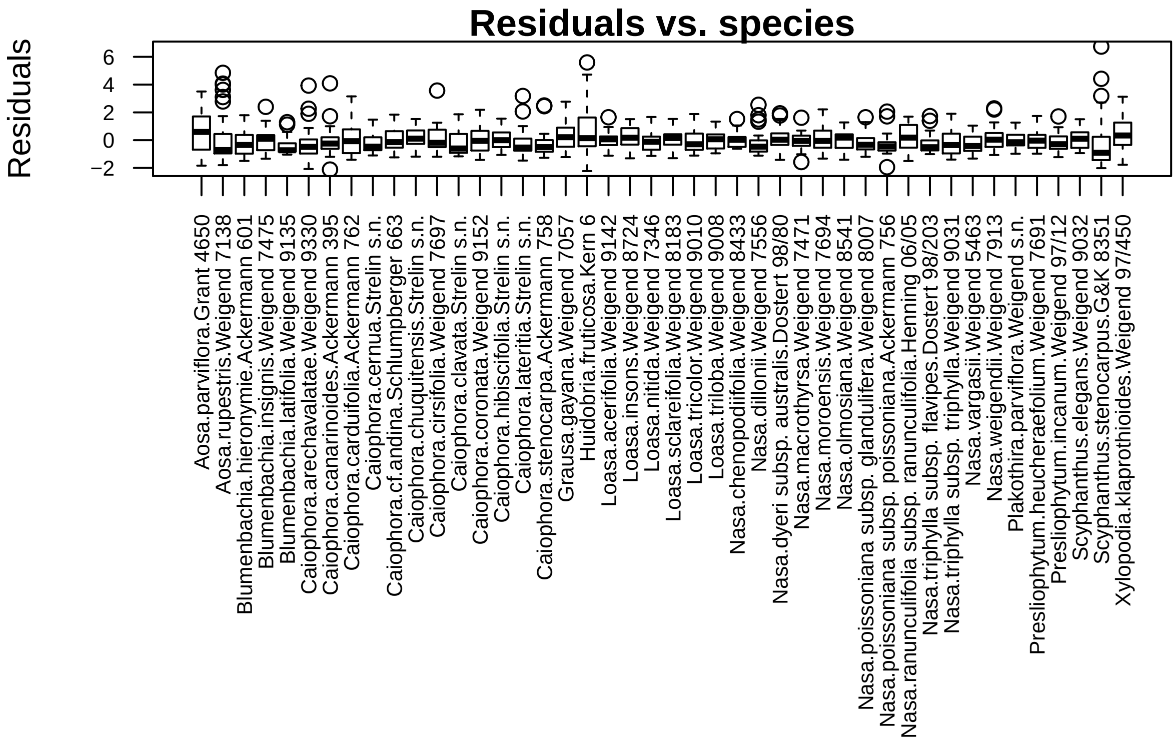

Figure 18: Plots of GAMM residuals against different predictors and factors.

In order to check for correlation effects imposed by pseudo-replication, we plot the residuals against the *species*, *impulse.period*, *PSG*, *impulse*, and *time.absolute*. We do not detect any strong patterns.

Case study: short-tongued bees

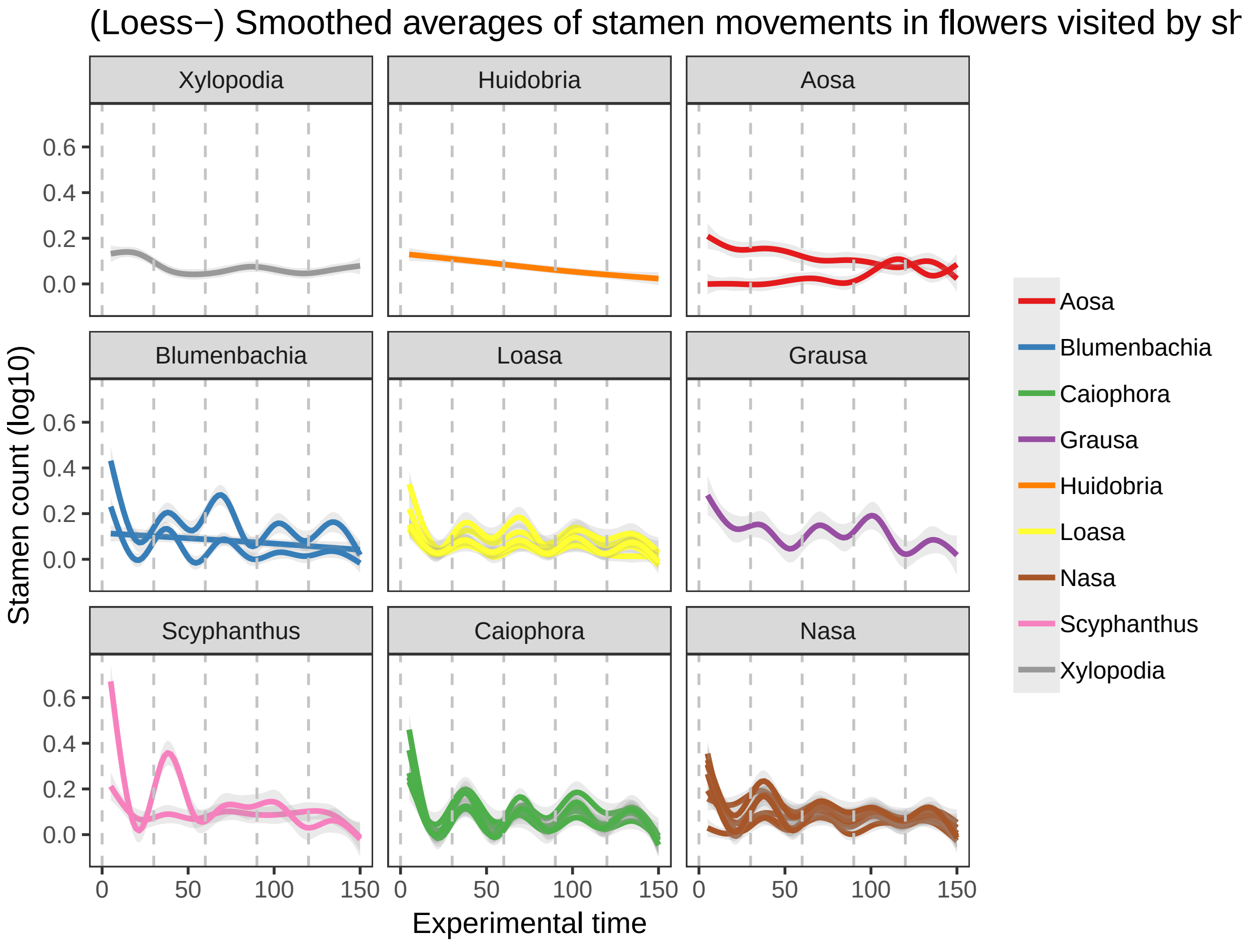

Figure 19: Differences in staminal movements during experimental time in reaction to manual stimulations of the floral organs in flowers. Dashed horizontal lines mark stimulations. First evaluation of staminal reaction occured 5 minutes after stimulus. Solid lines are averaged Loess-smooths colored for each genus. Shaded ribbons show 95% confidence intervals.

As most prominent pollination group and as only specialization sampled in multiple genera/groups, we choose short-tongued bees as model to analyze the adaptation of flowers to a defined syndrome over phylogenetic evolution. The plotted curves of stamen movements over the whole experimental time show stable patterns within *Caiophora*, *Loasa* and *Nasa*, while other genera exhibit more variable shapes.

GAMM

In order to concentrate on the differences between genera (and not to incorporate the variability within flowers of the same species) we aggregate stamen movements per species (as shown in previous graph) and use averages (*st*) as response. After model simplification and assessment we ended up with this (best) model:

Fixed: mean.stamens~s(impulse,k=6,by=Genus.order) + Genus.order + s(time.absolute)

Random: species

In addition we added an auto-correlation structure to account for pseudo-replication among *impulse.periods*:

correlation=corAR1(form=~1|impulse.period)

```
Family: poisson
Link function: log

Formula:
st ~ s(impulse, k = 6, by = Genus.order) + Genus.order + s(time.absolute)

Parametric coefficients:
                        Estimate Std. Error t value Pr(>|t|)
(Intercept)            -1.312885   0.854840  -1.536    0.125
Genus.orderHuidobria     0.007624   1.018112   0.007    0.994
Genus.orderAosa        -0.019902   1.049402  -0.019    0.985
Genus.orderBlumenbachia  0.116508   0.887307   0.131    0.896
Genus.orderLoasa       -0.488833   0.879973  -0.556    0.579
Genus.orderGrausa        0.249540   0.970667   0.257    0.797
Genus.orderScyphanthus   0.617728   0.904375   0.683    0.495
Genus.orderCaiophora    -0.261508   0.871592  -0.300    0.764
Genus.orderNasa         -0.061515   0.868795  -0.071    0.944

Approximate significance of smooth terms:
                                  edf Ref.df      F  p-value    
s(impulse):Genus.orderXylopodia    1.000  1.000  0.003  0.95486    
s(impulse):Genus.orderHuidobria    1.000  1.000  3.287  0.07020 .  
s(impulse):Genus.orderAosa         1.000  1.000  0.009  0.92623    
s(impulse):Genus.orderBlumenbachia 4.149  4.149 24.268  < 2e-16 ***
s(impulse):Genus.orderLoasa        1.000  1.000 49.666 3.66e-12 ***
s(impulse):Genus.orderGrausa       1.000  1.000  8.356  0.00394 ** 
s(impulse):Genus.orderScyphanthus  4.052  4.052 14.346 1.58e-11 ***
s(impulse):Genus.orderCaiophora    3.278  3.278 35.100  < 2e-16 ***
s(impulse):Genus.orderNasa         4.220  4.220 36.671  < 2e-16 ***
s(time.absolute)                   1.000  1.000  9.611  0.00200 ** 
---
Signif. codes:  0 '***' 0.001 '**' 0.01 '*' 0.05 '.' 0.1 ' ' 1

R-sq.(adj) =  0.573
  Scale est. = 1         n = 870
```

Model validation

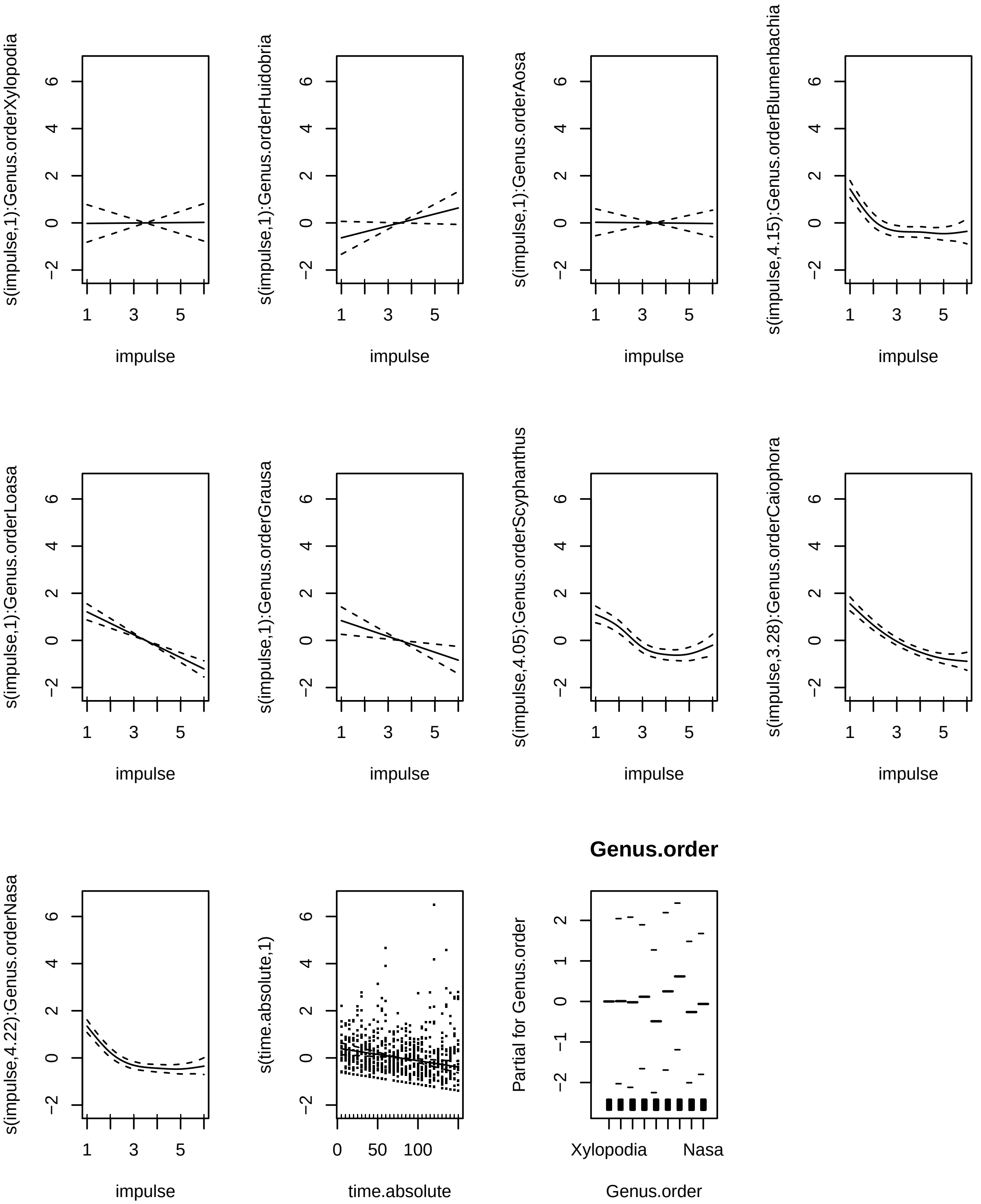

Figure 20: Smoother of each genus as estimated in GAMM for short tounged bees.

Table 3: Results of the phylosig() function ofr Blomberg's K and Pagel's lambda

Trait	K	K_p.value	lambda	lambda_p.value
X1st5min_thig	0.087	0.370	0.383	0.152
Sum_30min_thig	0.096	0.296	0.000	1.000
X.1st5min	0.091	0.288	0.636	0.001
control1st5min	0.100	0.252	0.154	0.263
control_sum30min	0.165	0.053	0.122	0.558
IncMove5min	0.064	0.606	0.298	0.284
X.MoveInc5min	0.057	0.696	0.046	0.772
IncMov30min	0.074	0.537	0.000	1.000
X.MoveInc30min	0.063	0.586	0.046	0.752
X.1st5minCont	0.089	0.357	0.077	0.565

Phylogenetic Signal

To test the phylogenetic signal of stamen movement patterns in Loasaceae, we calculated Blomberg's K and Pagel's λ using the 'phylosig()' function implemented in the R package phytools (Revell 2012). We used the implemented randomization test (1000 randomizations) or likelihood ratio test of the trait data-sets to generate a null distribution to test for significance of K or λ, respectively. Prior to testing for phylogenetic signal the out-groups used for constructing the phylogeny were trimmed from the tree with the drop.tip() function in the R package picante (Kembel et al. 2010). The resulting phylogenetic tree looks like this:

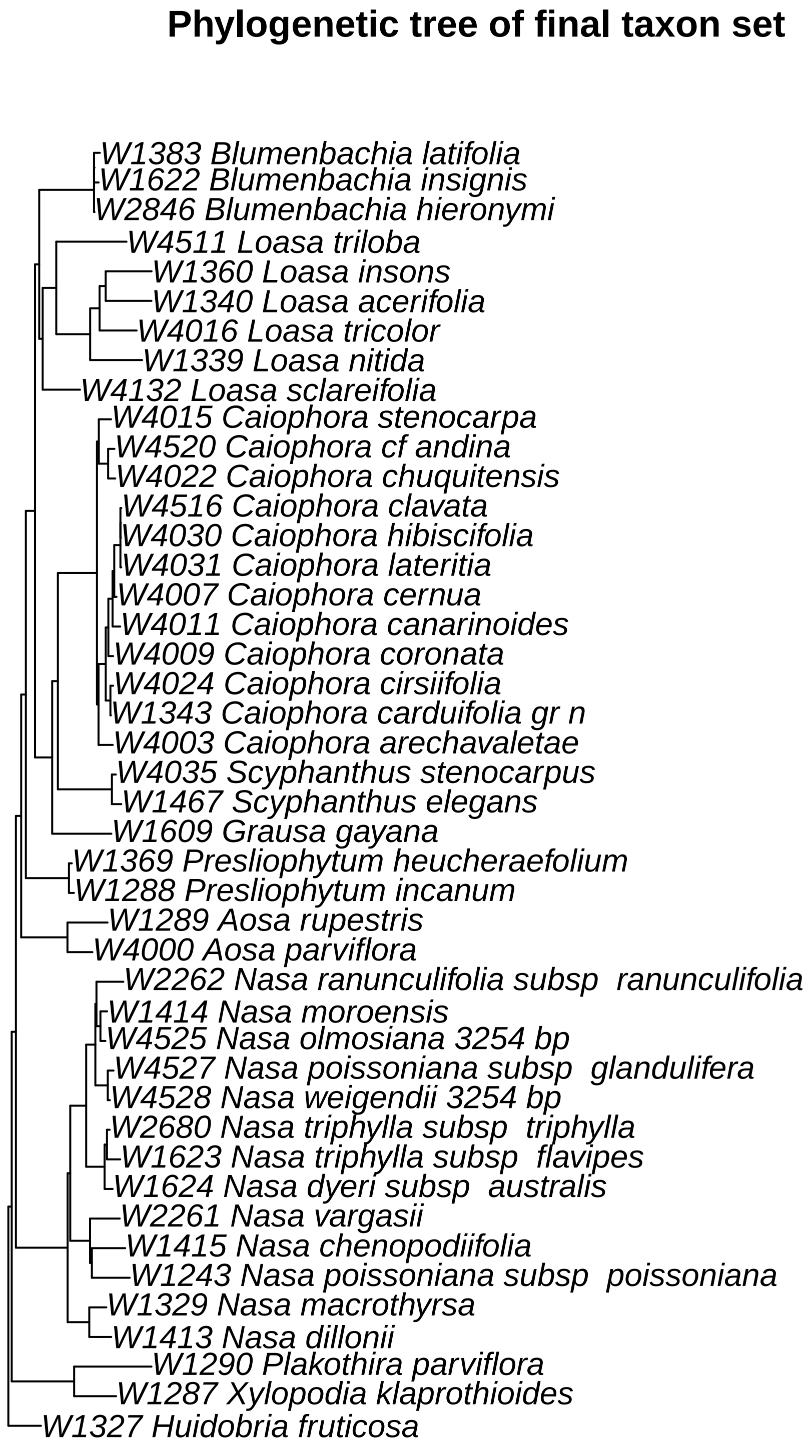

Figure 21: Phylogenetic tree of sampled species.

References

Benjamini, Yoav, and Yosef Hochberg. 1995. "Controlling the False Discovery Rate: A Practical and Powerful Approach to Multiple Testing." *Journal of the Royal Statistical Society. Series B (Methodological)* 57 (1). [Royal Statistical Society, Wiley]: 289–300. http://www.jstor.org/stable/2346101.

Jombart, Thibaut, François Balloux, and Stéphane Dray. 2010. "adephylo: New tools for investigating the phylogenetic signal in biological traits." *Bioinformatics* 26 (15): 1907–9. doi:10.1093/bioinformatics/btq292.

Kembel, Steven W., Peter D. Cowan, Matthew R. Helmus, William K. Cornwell, Helene Morlon, David D. Ackerly, Simon P. Blomberg, and Campbell O. Webb. 2010. "Picante: R tools for integrating phylogenies and ecology." *Bioinformatics* 26 (11): 1463–4. doi:10.1093/bioinformatics/btq166.

Revell, Liam J. 2012. "phytools: An R package for phylogenetic comparative biology (and other things)." *Methods in Ecology and Evolution* 3 (2): 217–23. doi:10.1111/j.2041-210X.2011.00169.x.

Wood, S N. 2011. "Fast stable restricted maximum likelihood and marginal likelihood estimation of semiparametric generalized linear models." *Journal of the Royal Statistical Society (B)* 73 (1): 3–36. http://onlinelibrary.wiley.com/doi/10.1111/j.1467-9868.2010.00749.x/abstract.

Zuur, Alain F. 2012. *A beginner's guide to generalized additive models with R.*

Zuur, Alain, F, Elena N Ieno, Neil Walker, Anatoly A Saveliev, and Graham M Smith. 2009. *Mixed EffectsModels and Extensions in Ecology with R.* Vol. 1. Springer. doi:10.1017/CBO9781107415324.004.
